# Untargeted Metabolomics Reveals the Protective Effect of a Traditional Chinese Herbal Decoction on Cisplatin-Induced Acute Kidney Injury

**DOI:** 10.1155/2020/8524132

**Published:** 2020-10-10

**Authors:** Yuyan Li, Xinhui Liu, Siqi Liu, Jiandong Lu, Jianping Chen, Guoliang Xiong, Shudong Yang, Shunmin Li

**Affiliations:** ^1^Department of Nephrology, Shenzhen Traditional Chinese Medicine Hospital, Guangzhou University of Chinese Medicine, Shenzhen, Guangdong, China; ^2^Shenzhen Key Laboratory of Hospital Chinese Medicine Preparation, Shenzhen Traditional Chinese Medicine Hospital, Guangzhou University of Chinese Medicine, Shenzhen, Guangdong, China

## Abstract

Our previous studies have demonstrated that Jian-Pi-Yi-Shen formula (JPYSF), a traditional Chinese herbal decoction, has a renoprotective effect in 5/6 nephrectomy-induced chronic kidney injury. However, the role and potential mechanisms of JPYSF in the treatment of acute kidney injury (AKI) remain unknown. This study was designed to test the beneficial effect of JPYSF in an AKI mouse model and to investigate the underlying mechanism by using metabolomics analysis. The AKI mouse model was induced by a single intraperitoneal injection of cisplatin at a dose of 20 mg/kg. The mice in the treatment group were pretreated orally with JPYSF (18.35 g/kg/d) for 5 days before cisplatin injection. Seventy-two hours after cisplatin injection, serum and kidney samples were collected for biochemical and histological examination. Ultra-high-performance liquid chromatography coupled with quadrupole time-of-ﬂight mass spectrometry (UHPLC-QTOF/MS) was applied to analyze metabolic profiling variations in the kidney. The results showed that pretreatment with JPYSF obviously reduced the levels of serum creatinine and blood urea nitrogen and alleviated renal pathological injury in AKI mice. Orthogonal partial least-squares discriminant analysis (OPLS-DA) score plot revealed a clear separation between the AKI and AKI + JPYSF group. A total of 68 and 87 significantly differentially expressed metabolites were identified in the kidney of AKI mice responding to JPYSF treatment in negative and positive ion mode, respectively. The pivotal pathways affected by JPYSF included vitamin B6 metabolism, alanine, aspartate and glutamate metabolism, lysine biosynthesis, and butanoate metabolism. In conclusion, JPYSF can protect the kidney from cisplatin-induced AKI, which may be associated with regulating renal metabolic disorders.

## 1. Introduction

Acute kidney injury (AKI) is a common disorder worldwide and is associated with high morbidity, mortality, and cost [1, 2]. Cisplatin is a widely used and highly effective anticancer drug for treating malignant tumor [3]. However, its clinical use is restricted given serious side effects, particularly AKI [4]. Although many mechanisms associated with cisplatin-induced AKI have been reported, the treatment strategy remains limited [5]. Traditional Chinese medicine (TCM) has been widely used for the treatment of AKI and its complications in China for a long time [6]. The therapeutic effects of TCM have been tested in animal models of AKI [7–11] and even in AKI patients [12]. Jian-Pi-Yi-Shen formula (JPYSF), a traditional Chinese herbal decoction, is composed of 8 herbs, including Astragali Radix, Atractylodis Macrocephalae Rhizoma, *Dioscoreae Rhizoma*, Cistanches Herba, Amomi Fructus Rotundus, Salviae Miltiorrhizae Radix et Rhizoma, Rhei Radix et Rhizoma, and Glycyrrhizae Radix et Rhizoma Praeparata cum Melle. According to previous studies, 4 main herbs of JPYSF may have a therapeutic effect on AKI, which is Astragali Radix [7], *Dioscoreae Rhizoma* [11], Salviae Miltiorrhizae Radix et Rhizoma [8], and Rhei Radix et Rhizoma [9]. Our previous studies have demonstrated the protective effect of JPYSF on delaying the progression of chronic kidney disease (CKD) and its complications [13–17]. However, the role and potential mechanisms of JPYSF in the treatment of AKI remain unclear.

Metabolomics aims to investigate the metabolic profiles of endogenous small molecular weight metabolites in the biological system responding to external stimuli [18]. TCM formulas are usually composed of many herbs, which makes it difficult to fully understand their therapeutic mechanisms. Metabolomics strategy is beneficial to clarify the connotation of TCM theory [19] and has been successfully applied in many TCM-related research fields, such as therapeutic mechanisms elucidation [20, 21], drug toxicity evaluation [22], and disease diagnosis [23]. Hence, metabolomics provides an approach to achieve a comprehensive and systematic understanding of the therapeutic mechanisms of TCM formulas.

In the present study, cisplatin-induced AKI was established by a single intraperitoneal injection of cisplatin at a dose of 20 mg/kg [24]. Renal function parameters and renal pathology were used to evaluate the characteristics of AKI and efficacy of JPYSF treatment. Moreover, ultra-high-performance liquid chromatography coupled with quadrupole time-of-flight mass spectrometry- (UHPLC-QTOF/MS-) based untargeted metabolomics approach was used to analyze the changes in renal metabolites and the response to JPYSF treatment in cisplatin-induced AKI mouse model.

## 2. Materials and Methods

### 2.1. Chemicals and Materials

Cisplatin was purchased from Sigma-Aldrich (St. Louis, MO, USA). The herbal composition and dosage of JPYSF are shown in Table 1. All herbs were purchased from Shenzhen Huahui Pharmaceutical Co., Ltd. (Shenzhen, China). JPYSF water extract was prepared as previously described [13, 14]. Briefly, the raw herbs weighed according to the dose ratio were boiled twice with ddH_2_O and centrifuged at 13,000 rpm for 10 min. The supernatant was separated and dried into powder with a freeze dryer and stored at –80°C. High-performance liquid chromatography-mass spectrometry (HPLC-MS) analysis was conducted to confirm the quality of the JPYSF extract, as indicated in Supplementary Figure 1. JPYSF water extract powder was redissolved in ddH_2_O for intragastric administration.

### 2.2. Animals and Treatment

All animal experiments were conducted with protocols approved by the Ethics Committee of Shenzhen Traditional Chinese Medicine Hospital, Guangzhou University of Chinese Medicine. Male 8-week-old C57BL/6 mice were obtained from Nanjing Biomedical Research Institute of Nanjing University (Jiangsu, China, permission no. SCXK (SU) 2018-0008). After one week of acclimatization, all mice were randomly divided into three groups: (1) control group (*n* = 6), (2) AKI group (*n* = 6), and (3) AKI + JPYSF group (*n* = 6). AKI was induced by a single intraperitoneal (i.p.) injection of cisplatin at the dose of 20 mg/kg. The mice in the AKI + JPYSF group were pretreated orally with JPYSF (18.35 g/kg/d) for 5 days before cisplatin injection. Seventy-two hours after cisplatin injection, all mice were sacrificed, and blood samples and kidneys were collected for further analysis.

### 2.3. Serum Biochemical Analysis

Serum samples were collected from blood by centrifugation at 1,000 rpm for 10 min. The levels of serum creatinine (Scr) and blood urea nitrogen (BUN) were measured by using creatinine serum detection kit and BUN detection kit (StressMarq Biosciences, British Columbia, Canada), respectively, according to the manufacturer's instructions.

### 2.4. Histological Analysis

To evaluate the histological injury, periodic acid-Schiff (PAS) staining was performed and analyzed in a blind procedure. Five fields for each kidney slide and three mice in each group were scored for pathological injury. The tubular injury was graded by a semiquantitative score from 0 to 4: 0, normal tubules; 1, injury affecting less than 25% of tubules; 2, injury affecting 25% to 50% of tubules; 3, injury affecting 50% to 75% of tubules; and 4, injury affecting more than 75% of tubules [25].

### 2.5. Sample Preparation for UHOLC-QTOF/MS

For sample preparation, 25 mg of renal tissue was added with 1000 *μ*L extract solution (acetonitrile: methanol: water = 2 : 2 : 1, v/v). After 30 s vortex, the samples were homogenized at 35 Hz for 4 min and sonicated for 5 min in an ice-water bath for 2 times. Then, the samples were centrifuged at 10,000 rpm for 15 min at 4°C. The supernatant (825 *μ*L) was transferred to a fresh tube and dried in a vacuum concentrator at 37°C. The residue was reconstituted in 200 *μ*L of 50% acetonitrile by sonication on ice for 10 min. The constitution was then centrifuged at 13,000 rpm for 15 min at 4°C, and 75 *μ*L of supernatant was transferred to a fresh glass vial for LC/MS analysis. The quality control (QC) sample was prepared by mixing an equal aliquot of the supernatants from each group. The overlapping total ion chromatograms (TICs) of QC samples demonstrated the acceptable variations occurred during the large-scale sample analysis (Supplementary Figure 2).

### 2.6. UHPLC-QTOF/MS Analysis

The UHPLC separation was carried out using a 1290 Infinity Series UHPLC System (Agilent Technologies), equipped with a UPLC BEH Amide column (2.1 × 100 mm, 1.7 *μ*m, Waters). The mobile phase consisted of 25 mmol/L ammonium acetate and 25 mmol/L ammonia hydroxide in water (pH = 9.75) (A) and acetonitrile (B). The analysis was carried with elution gradient as follows: 0–0.5 min, 95% B; 0.5–7.0 min, 95%–65% B; 7.0–8.0 min, 65%–40% B; 8.0–9.0 min, 40% B; 9.0–9.1 min, 40%–95% B; and 9.1–12.0 min, 95% B. The column temperature was 25°C. The auto-sampler temperature was 4°C, and the injection volume was 1 *μ*L (pos) or 1 *μ*L (neg), respectively.

The TripleTOF 6600 mass spectrometry (AB Sciex) was used for its ability to acquire MS/MS spectra on an information-dependent basis (IDA) during an LC/MS experiment. In this mode, the acquisition software (Analyst TF 1.7, AB Sciex) continuously evaluates the full scan survey MS data as it collects and triggers the acquisition of MS/MS spectra depending on preselected criteria. In each cycle, the most intensive 12 precursor ions with intensity above 100 were chosen for MS/MS at collision energy (CE) of 30 eV. The cycle time was 0.56 s. ESI source conditions were set as follows: gas 1 as 60 psi, gas 2 as 60 psi, curtain gas as 35 psi, source temperature as 600°C, declustering potential as 60 V, and ion spray voltage floating (ISVF) as 5000 V or −4000 V in positive or negative mode, respectively.

### 2.7. Data Processing

MS raw data (.wiff) files were converted to the mzXML format by ProteoWizard and processed by R package XCMS (version 3.2). The process includes peak deconvolution, alignment, and integration. Minfrac and cutoff are set as 0.5 and 0.6, respectively. In-house MS2 database was applied for metabolite identification. The SIMCA software (V15.0.2, Sartorius Stedim Data Analytics AB, Umeå, Sweden) was used for principal components analysis (PCA) and orthogonal partial least-squares discriminant analysis (OPLS-DA). The potential metabolic biomarkers were evaluated according to variable importance in projection (VIP) > 1.0 from OPLS-DA and *P* value < 0.05 from Student's *t*-test. Pathway analysis was based on the Kyoto Encyclopedia of Genes and Genomes (KEGG) (http://www.genome.jp/kegg/) and MetaboAnalyst (http://www.metaboanalyst.ca/).

### 2.8. Statistical Analysis

Results were presented as the mean ± standard error of the mean (SEM). SPSS 16.0 statistics software (SPSS Inc., Chicago, IL, USA) was used for statistical analyses. Statistically significant differences were determined by one-way ANOVA followed by Tukey's multiple comparisons test. The significance threshold was set at *P* < 0.05.

## 3. Results

### 3.1. JPYSF Alleviated Cisplatin-Induced AKI in Mice

**Figure 1:**
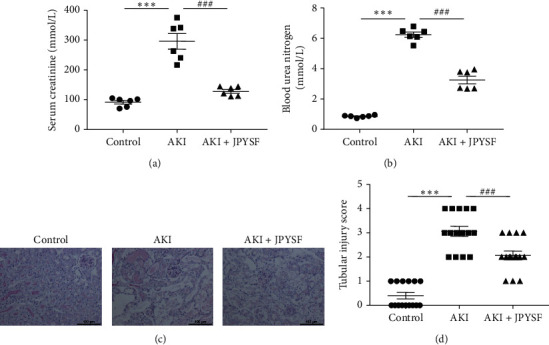
Effects of JPYSF on cisplatin-induced AKI. (a) Serum creatinine (*n* = 6). (b) Blood urea nitrogen (*n* = 6). (c) PAS staining. (d) Tubular injury score (five microscopic fields of each mouse and three mice in each group). Data are presented as the means ± SEM (^*∗∗∗*^*P* < 0.001 compared with the control group; ^###^*P* < 0.001 compared with the AKI group).

After 72 h of cisplatin injection, the mice in the AKI group showed a significant increase in the levels of Scr and BUN. Strikingly, the enhanced Scr and BUN levels were significantly reversed by JPYSF pretreatment (Figures 1(a) and 1(b)). Parallel to the deterioration of renal function, PAS staining in AKI mice showed obvious tubular injury manifested as tubular necrosis, loss of the brush border, and tubular dilation. In agreement with improved renal function, tubular injury was markedly attenuated in the AKI + JPYSF group (Figures 1(c) and 1(d)). These data demonstrated that JPYSF pretreatment alleviated cisplatin-induced AKI in mice.

### 3.2. Renal Metabolic Profiles Change in Cisplatin-Induced AKI

**Figure 2:**
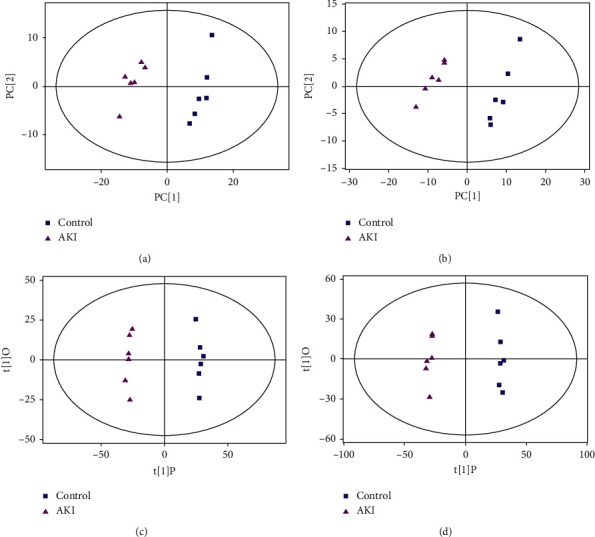
The score plots from PCA and OPLS-DA model based on metabolic profiles. PCA score plot for the AKI versus control group in negative (a) and positive (b) ion mode. OPLS-DA score plot for the AKI versus control group in negative (c) and positive (d) ion mode.

**Figure 3:**
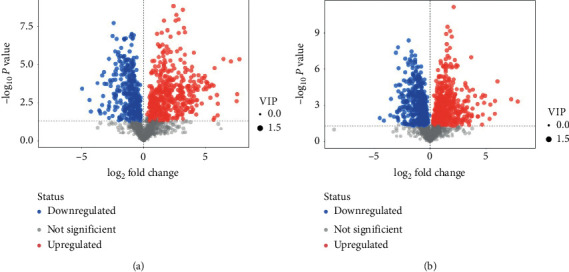
Volcano plots of differential metabolites. Comparison between the AKI and the control group in negative (a) and positive (b) ion mode.

**Figure 4:**
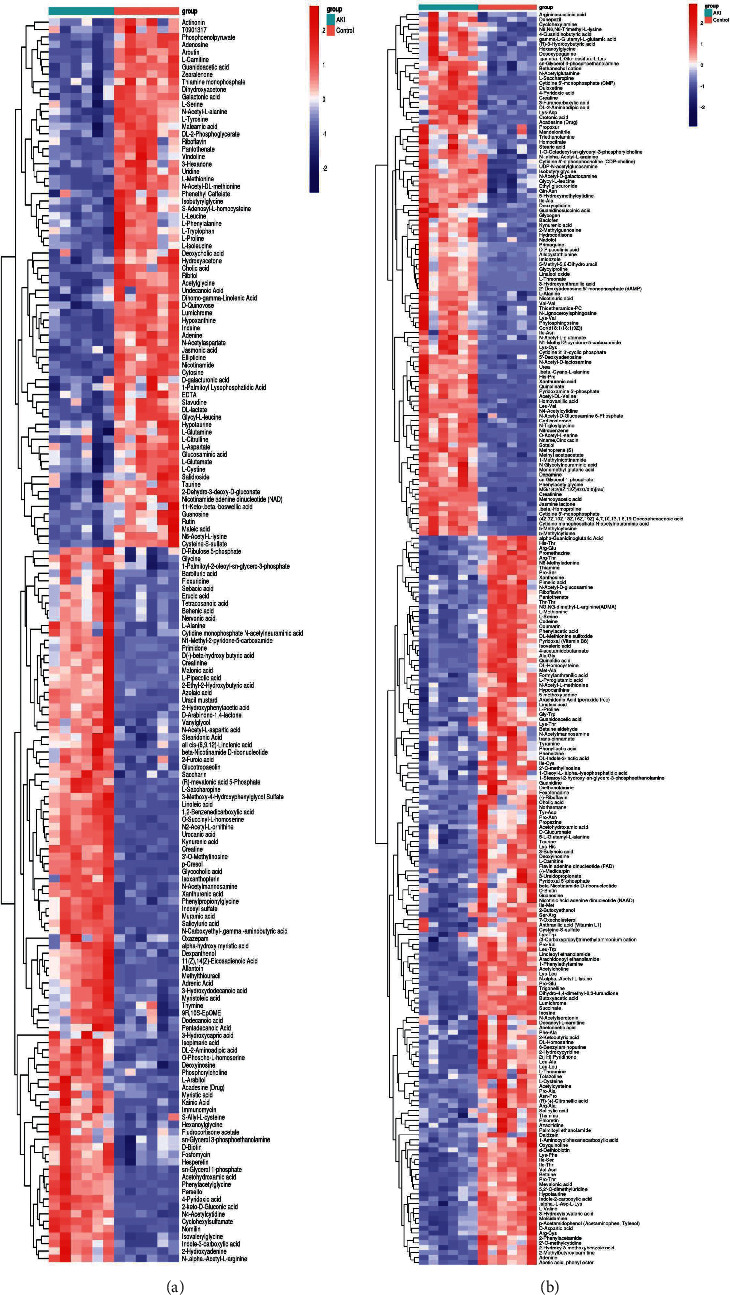
Heatmap of hierarchical clustering analysis of the differential metabolites. Comparison between the AKI and the control group in negative (a) and positive (b) ion mode.

**Figure 5:**
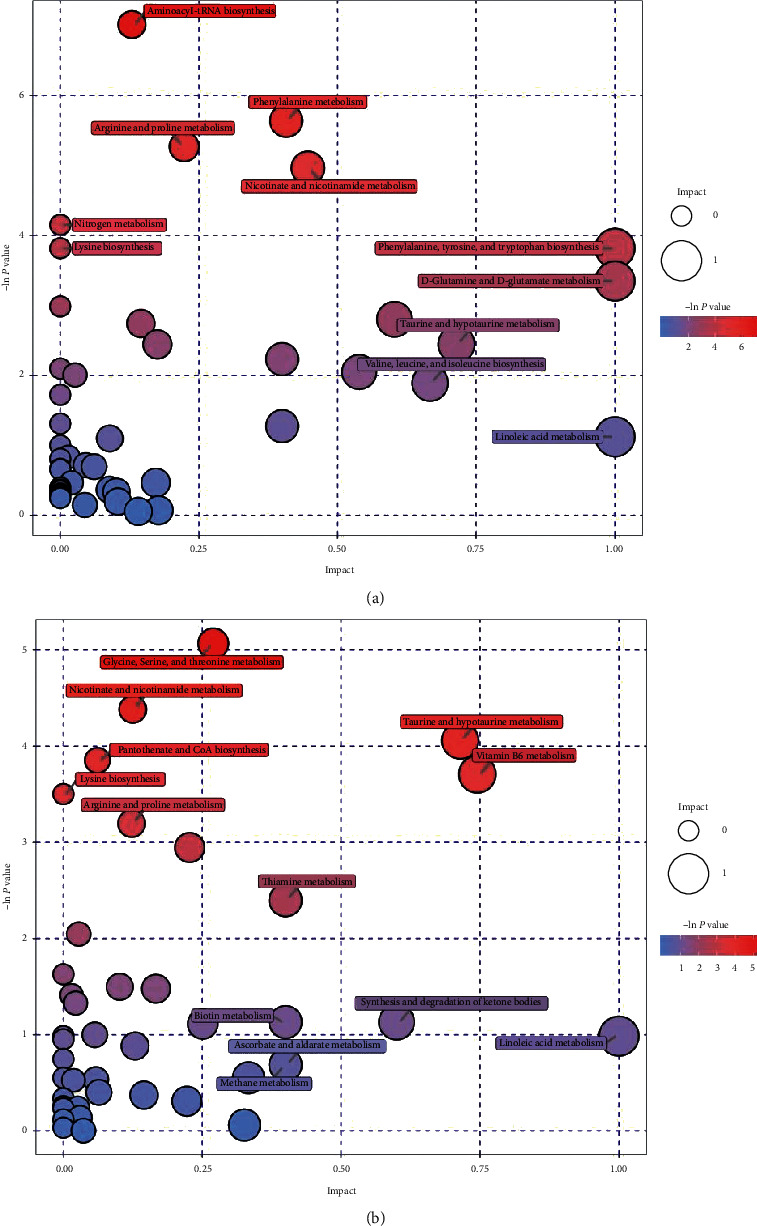
Bubble plots of the metabolic pathway analysis. The main metabolic pathways changed by AKI in negative (a) and positive (b) ion mode.

PCA is an unsupervised pattern recognition method. In the PCA score scatter plots, there were distinctly separated clusters between the control and the AKI group in both negative ion mode (Figure 2(a)) and positive ion mode (Figure 2(b)). Similar findings were found in the score plots of OPLS-DA (Figures 2(c) and 2(d)), a more reliable pattern recognition method. Potential biomarkers were screened according to *P* value of the *t*-test < 0.05 and VIP in the OPLS-DA model > 1.0. In total, 167 and 256 significantly differentially expressed metabolites were identified in the kidney in negative and positive ion mode, respectively (Supplementary Tables 1 and 2). The significantly differential metabolites were visualized by using volcano plots (Figures 3(a) and 3(b)). The figures showed change trends of differential metabolites in AKI mice versus control mice. Hierarchical clustering analysis of differential metabolites is shown in Figures 4(a) and 4(b). In negative ion mode, the main metabolic pathways significantly affected by AKI included phenylalanine, tyrosine and tryptophan biosynthesis, D-glutamine and D-glutamate metabolism, nicotinate and nicotinamide metabolism, and phenylalanine metabolism (Figure 5(a)). In positive ion mode, the main metabolic pathways significantly affected by AKI included vitamin B6 metabolism, taurine and hypotaurine metabolism, glycine, serine and threonine metabolism, and nicotinate and nicotinamide metabolism (Figure 5(b)).

### 3.3. The Influence of JPYSF on the Renal Metabolic Profiles in Cisplatin-Induced AKI

**Figure 6:**
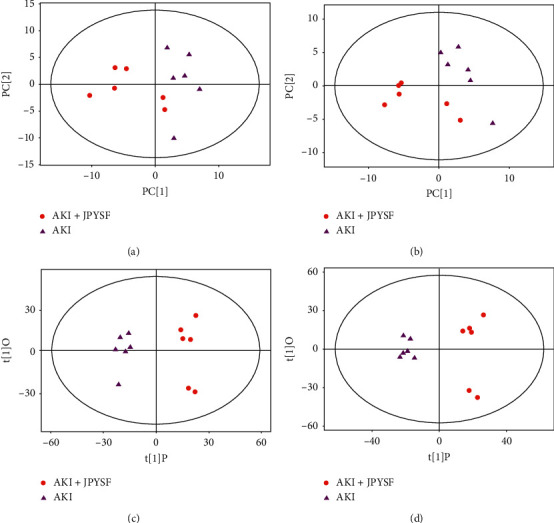
The score plots from PCA and OPLS-DA model based on metabolic profiles. PCA score plot for the AKI + JPYSF versus AKI group in negative (a) and positive (b) ion mode. OPLS-DA score plot for the AKI + JPYSF versus AKI group in negative (c) and positive (d) ion mode.

**Figure 7:**
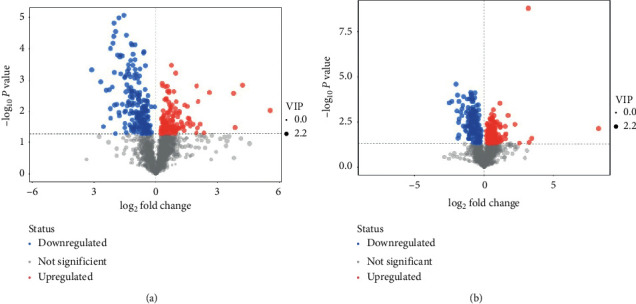
Volcano plots of differential metabolites. Comparison between the AKI + JPYSF and the AKI group in negative (a) and positive (b) ion mode.

**Figure 8:**
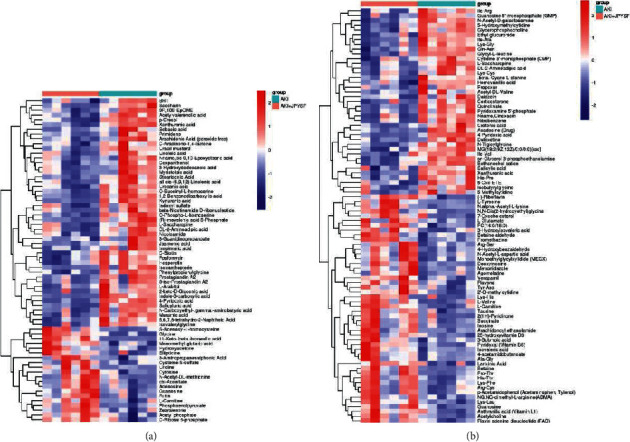
Heatmap of hierarchical clustering analysis of the differential metabolites. Comparison between the AKI + JPYSF and the AKI group in negative (a) and positive (b) ion mode.

**Figure 9:**
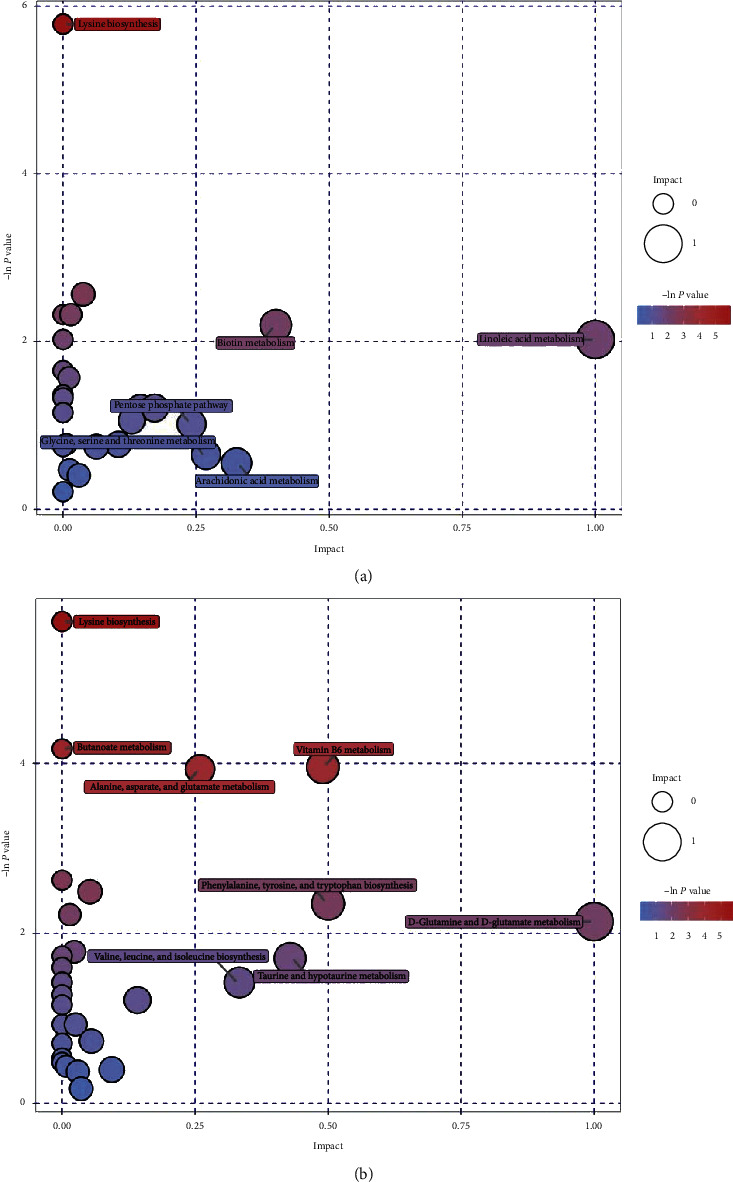
Bubble plots of the metabolic pathway analysis. The main metabolic pathways responded to JPYSF treatment in negative (a) and positive (b) ion mode.

PCA score plots did not separate the AKI + JPYSF group from the AKI group very well (Figures 6(a) and 6(b)). To improve the classification, the OPLS-DA model was performed, and the samples in the AKI + JPYSF group and the AKI group were clearly separated (Figures 6(c) and 6(d)). A total of 68 and 87 significantly differentially expressed metabolites were identified in the kidney of AKI + JPYSF mice versus AKI mice in negative and positive ion mode, respectively (Supplementary Tables 3 and 4). The change trends of these metabolites were illustrated by volcano plots (Figures 7(a) and 7(b)). Heatmap of hierarchical clustering analysis for the AKI + JPYSF group versus AKI group is shown in Figures 8(a) and 8(b). In negative ion mode, the main metabolic pathway significantly affected by JPYSF treatment was lysine biosynthesis (Figure 9(a)). In positive ion mode, the main metabolic pathways significantly affected by JPYSF treatment included vitamin B6 metabolism, alanine, aspartate and glutamate metabolism, lysine biosynthesis, and butanoate metabolism (Figure 9(b)).

## 4. Discussion

In the present study, we investigated the efficacy and potential mechanisms of JPYSF in treating cisplatin-induced AKI. The results showed that JPYSF markedly reduced the levels of Scr and BUN and improved tubular injury in AKI mice. Moreover, renal metabolites partially restored the response to JPYSF treatment in AKI mice.

AKI is characterized by a rapid decline of renal function and can lead to the development of CKD or end-stage renal disease (ESRD). Numerous causes can induce AKI, including renal ischemia-reperfusion injury (IRI), nephrotoxic insults, and sepsis [26]. In recent years, increasing evidence reveals the beneficial role of TCM in improving AKI, and many mechanisms have been illustrated [6]. Several TCM formulas have been tested in the treatment of AKI. Dahuang Fuzi decoction could alleviate adenine-induced tubular epithelial apoptosis and renal damage through the suppression of TGF-*β*1-JNK pathway activation [9]. Li et al. reported that Huang-Lian-Jie-Du decoction effectively inhibited LPS-induced AKI in mice by inhibiting NF-*κ*B and MAPK activation and activating the Akt/HO-1 pathway [10]. Hsu et al. found that Zhibai Dihuang Wan attenuated gentamicin-induced AKI by limiting caspase-3 activation [11]. The renoprotective effect of JPYSF on delaying CKD progression has been reported by our group via using 5/6 nephrectomized rats. In this study, we found that JPYSF pretreatment could attenuate cisplatin-induced AKI, which further confirmed the protective effect of JPYSF on the kidney.

The main metabolic pathways significantly affected by cisplatin were amino acids and cofactor metabolism, which is consistent with a previous study reported by Irie et al. [27]. Of note, nicotinate and nicotinamide metabolism was identified in both negative and positive ion mode (Figures 5(a) and 5(b)). Nicotinamide adenine dinucleotide (NAD) is an important cofactor involved in numerous physiological processes, including metabolism, posttranslational protein modification, and DNA repair [28]. Recent studies revealed that impaired NAD biosynthesis was associated with the severity of AKI in humans [29] and animal models [30]. Therefore, NAD augmentation may be beneficial in the prevention and treatment of AKI. The most important metabolic pathway response to JPYSF was vitamin B6 metabolism (Figures 9(a) and 9(b)). The active form of vitamin B6, pyridoxal 5′-phosphate (PLP), serves as a cofactor in more than 150 enzymatic reactions [31]. Plasma PLP predicts the risk of chronic diseases such as cardiovascular disease, diabetes, and some cancers and is inversely associated with numerous inflammatory markers in clinical and population-based studies [32]. The majority of patients with CKD can develop vitamin B6 deficiency from many causes [33]. Abnormal vitamin B6 level has been reported to be associated with diabetic kidney disease [34], kidney stones [35], and kidney cancer [36]. Skrypnyk et al. found that treatment with pyridoxamine, a structural analog of vitamin B6, reduced short- and long-term injury, fibrosis, and renal functional recovery after ischemia-reperfusion AKI [37]. The role of vitamin B6 metabolism in the pathogenesis of AKI needs further investigation.

In conclusion, JPYSF significantly alleviated cisplatin-induced AKI in mice, which might be associated with regulating renal metabolic disorders.

## Figures and Tables

**Table 1:** The herbal composition and dosage of JPYSF.

Botanical name	Herbal name	Chinese name	Voucher number	Dosage
*Astragalus membranaceus* (Fisch.) Bge. var. *mongholicus* (Bge.) Hsiao	Astragali Radix	Huang Qi	2010015Z	30 g
*Atractylodes macrocephala* Koidz.	Atractylodis Macrocephalae Rhizoma	Bai Zhu	2010024ZZ	10 g
*Dioscorea opposita* Thunb.	Dioscoreae Rhizoma	Shan Yao	2010037Z	30 g
*Cistanche deserticola* Y.C. Ma	Cistanches Herba	Rou Cong Rong	2040056Z	10 g
*Amomum kravanh* Pierre ex Gagnep.	Amomi Fructus Rotundus	Dou Kou	202086Z	10 g
*Salvia miltiorrhiza* Bunge.	Salviae Miltiorrhizae Radix et Rhizoma	Dan Shen	2010006Z	15 g
*Rheum palmatum* L.	Rhei Radix et Rhizoma	Da Huang	2010040Z	10 g
*Glycyrrhiza uralensis* Fisch.	Glycyrrhizae Radix et Rhizoma Praeparata cum Melle	Zhi Gan Cao	2010008ZZ	6 g

## Data Availability

The data used to support the findings of this study are available from the corresponding author upon request.

## Supplementary Materials

Supplementary Materials Supplementary Figure 1: the HPLC-MS profile of JPYSF extract. Supplementary Figure 2: the overlapping typical total ion chromatograms (TICs) of QC samples obtained from LC-MS in negative and positive modes. Supplementary Table 1: significantly differentially expressed metabolites in the kidney of AKI mice versus control mice in negative ion mode. Supplementary Table 2: significantly differentially expressed metabolites in the kidney of AKI mice versus control mice in positive ion mode. Supplementary Table 3: significantly differentially expressed metabolites in the kidney of AKI + JPYSF mice versus AKI mice in negative ion mode. Supplementary Table 4: significantly differentially expressed metabolites in the kidney of AKI + JPYSF mice versus AKI mice in positive ion mode.
