# Supplementary material for: Untargeted Metabolomics Reveals the Protective Effect of a Traditional Chinese Herbal Decoction on Cisplatin-Induced Acute Kidney Injury
Source: Evid Based Complement Alternat Med. 2020 Oct 10;2020:8524132. doi: 10.1155/2020/8524132 (PMC7569447; doi:10.1155/2020/8524132)
Supplement: Supplementary Materials — Supplementary Figure 1: the HPLC-MS profile of JPYSF extract. Supplementary Figure 2: the overlapping typical total ion chromatograms (TICs) of QC samples obtained from LC-MS in negative and positive modes. Supplementary Table 1: significantly differentially expressed metabolites in the kidney of AKI mice versus control mice in negative ion mode. Supplementary Table 2: significantly differentially expressed metabolites in the kidney of AKI mice versus control mice in positive ion mode. Supplementary Table 3: significantly differentially expressed metabolites in the kidney of AKI + JPYSF mice versus AKI mice in negative ion mode. Supplementary Table 4: significantly differentially expressed metabolites in the kidney of AKI + JPYSF mice versus AKI mice in positive ion mode. [file 8524132.f1.docx]

**Supplementary Figures**

**
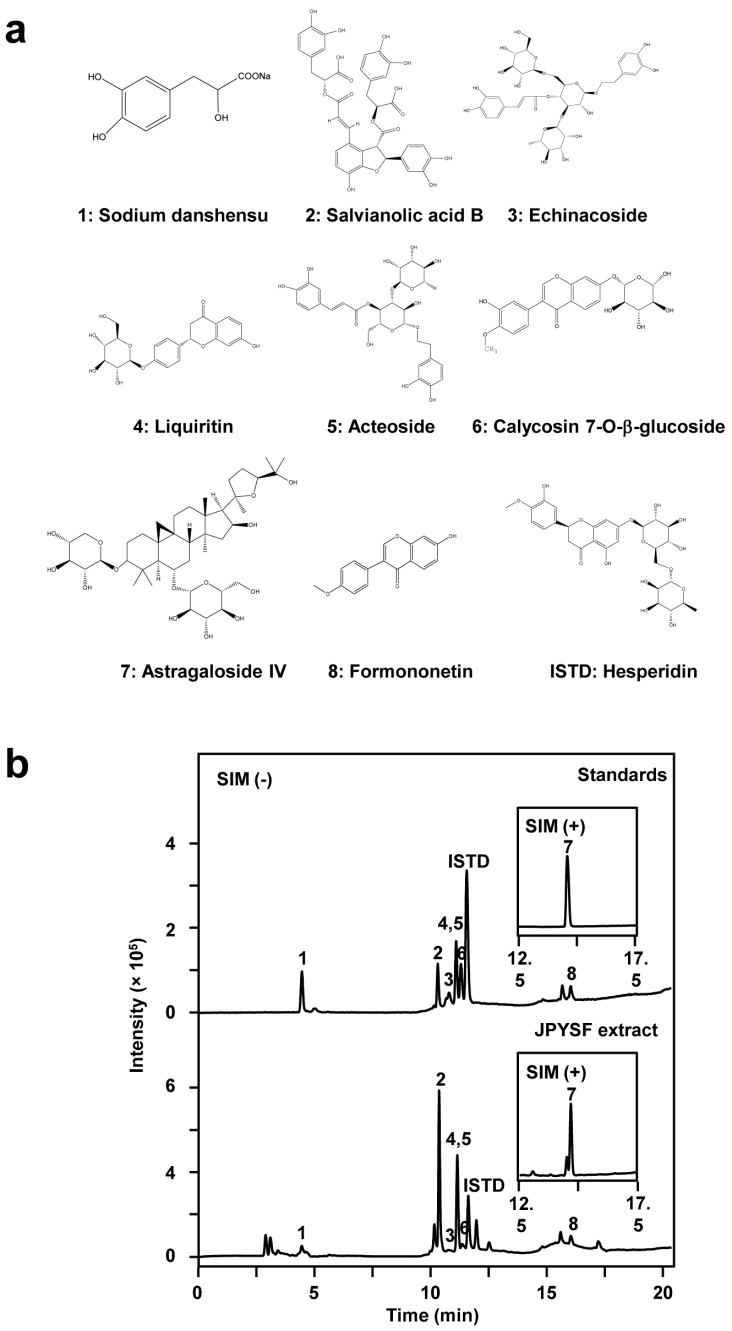
**

**Supplementary Figure 1. The HPLC-MS profile of JPYSF extract.** (a) Structures of chemical markers analyzed in JPYSF extract, including sodium danshensu (1), salvianolic acid B (2), echinacoside (3), liquiritin (4), acteoside (5), calycosin 7-O-β-glucoside (6), astragaloside IV (7), formononetin (8), and hesperidin (internal standard, ISTD). (b) The representative HPLC-MS chromatograms of mixed standards and JPYSF extract. (Liu X, *et al*. BMC Complement Altern Med, 2018; 18: 340.)

**a**


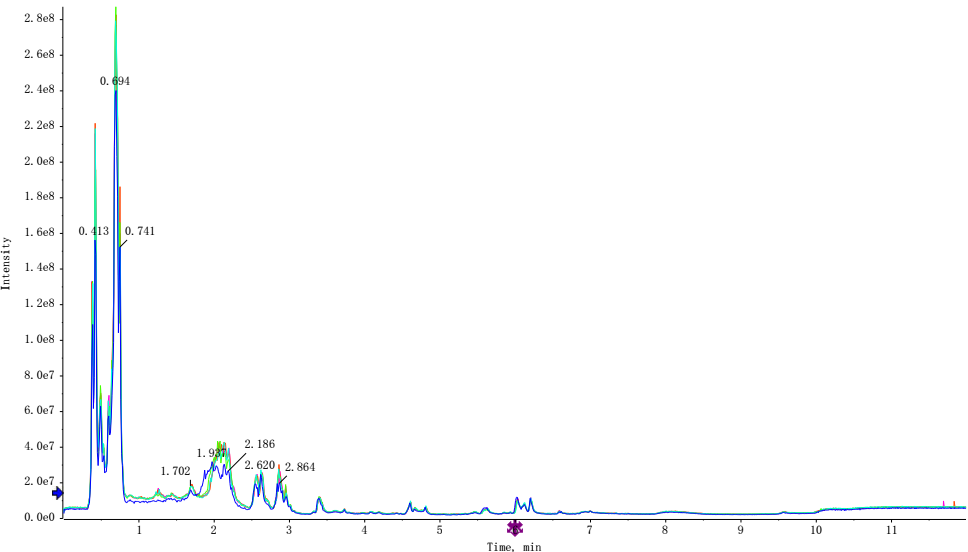


**b**

**
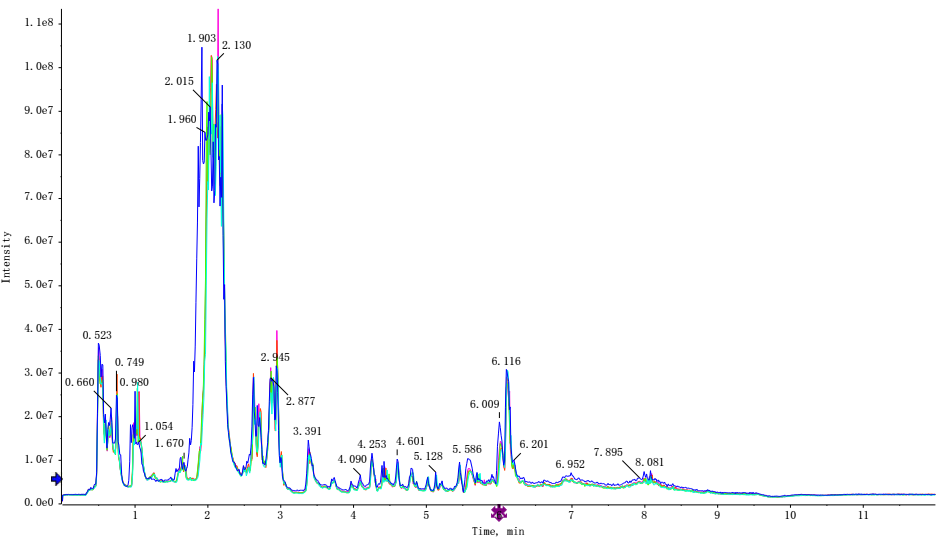
**

**Supplementary Figure 2. The overlapping typical total ion chromatograms (TICs) of QC samples obtained from LC-MS in negative (a) and positive (b) modes.**

**Supplementary Tables**

**Supplementary Table 1.** Significantly differentially expressed metabolites in the kidney of AKI mice *versus* control mice in negative ion mode

| NO. | Metabolites | m/z | Rt (min) | VIP | P Value | Fold Change |
| --- | --- | --- | --- | --- | --- | --- |
| 1 | (R)-mevalonic acid 5-Phosphate | 265.0837 | 124.3605 | 1.495067 | 0.002191 | 15.58339 |
| 2 | 1,2-Benzenedicarboxylic acid | 165.0179 | 123.486 | 1.533953 | 0.000314 | 8.665324 |
| 3 | 11(Z),14(Z)-Eicosadienoic Acid | 307.2611 | 41.699 | 1.38415 | 0.002614 | 1.477768 |
| 4 | 11-Keto-.beta.-boswellic acid | 469.3262 | 48.4455 | 1.1416 | 0.03366 | 0.474525 |
| 5 | 1-Palmitoyl Lysophosphatidic Acid | 409.2316 | 177.953 | 1.011408 | 0.016509 | 0.769718 |
| 6 | 1-Palmitoyl-2-oleoyl-sn-glycero-3-phosphate | 673.4796 | 133.9045 | 1.045403 | 0.006677 | 1.556623 |
| 7 | 2-Dehydro-3-deoxy-D-gluconate | 177.039 | 373.252 | 1.214608 | 0.001418 | 0.639498 |
| 8 | 2-Ethyl-2-Hydroxybutyric acid | 131.0703 | 134.748 | 1.405759 | 0.015441 | 3.537469 |
| 9 | 2-Furoic acid | 128.0341 | 97.266 | 1.240053 | 0.002803 | 1.715949 |
| 10 | 2-Hydroxyadenine | 188.0006 | 96.83 | 1.350516 | 0.006858 | 2.406393 |
| 11 | 2-Hydroxyphenylacetic acid | 151.0392 | 33.177 | 1.419251 | 0.002972 | 3.271162 |
| 12 | 2-keto-D-Gluconic acid | 210.0588 | 68.921 | 1.432855 | 0.000229 | 7.307161 |
| 13 | 3-Hexanone | 137.0349 | 158.9815 | 1.24293 | 0.003192 | 0.67394 |
| 14 | 3-Hydroxycapric acid | 209.1169 | 107.6825 | 1.091562 | 0.038498 | 1.89638 |
| 15 | 3-Hydroxydodecanoic acid | 197.1533 | 46.47 | 1.353324 | 0.004247 | 2.273905 |
| 16 | 3-Methoxy-4-Hydroxyphenylglycol Sulfate | 263.0211 | 39.164 | 1.503324 | 0.002027 | 13.88624 |
| 17 | 3'-O-Methylinosine | 281.0866 | 141.418 | 1.466154 | 5.86E-06 | 1.643619 |
| 18 | 4-Pyridoxic acid | 182.0445 | 39.163 | 1.537318 | 5.67E-05 | 30.71457 |
| 19 | 9R,10S-EpOME | 295.225 | 46.226 | 1.217972 | 0.002726 | 1.597464 |
| 20 | Acadesine (Drug) | 257.0864 | 186.113 | 1.162668 | 0.013701 | 2.518819 |
| 21 | Acetohydroxamic acid | 74.02437 | 164.753 | 1.512616 | 0.000719 | 9.001469 |
| 22 | Acetylglycine | 233.0763 | 299.319 | 1.449789 | 8.63E-06 | 0.477462 |
| 23 | Actinonin | 384.2522 | 99.0725 | 1.02113 | 0.013329 | 0.44948 |
| 24 | Adenine | 134.0467 | 149.35 | 1.467043 | 0.000286 | 0.307944 |
| 25 | Adenosine | 304.0501 | 204.686 | 1.460736 | 9.05E-06 | 0.58347 |
| 26 | Adrenic Acid | 331.262 | 40.384 | 1.367413 | 0.002388 | 1.619456 |
| 27 | all cis-(6,9,12)-Linolenic acid | 277.215 | 43.02 | 1.252375 | 0.003615 | 1.636822 |
| 28 | Allantoin | 157.0354 | 172.676 | 1.520214 | 0.001458 | 9.635498 |
| 29 | alpha-hydroxy myristic acid | 243.1945 | 46.8525 | 1.259545 | 0.00779 | 1.851886 |
| 30 | Arbutin | 293.0671 | 204.686 | 1.494161 | 3.37E-06 | 0.676305 |
| 31 | Azelaic acid | 187.096 | 303.54 | 1.387247 | 0.002124 | 3.817118 |
| 32 | Barbituric acid | 187.0357 | 114.833 | 1.227891 | 0.013494 | 2.676481 |
| 33 | Behenic acid | 339.323 | 39.869 | 1.200354 | 0.017962 | 3.302418 |
| 34 | beta-Nicotinamide D-ribonucleotide | 356.0337 | 25.774 | 1.490461 | 0.006213 | 14.62956 |
| 35 | Cholic acid | 407.2754 | 175.821 | 1.416115 | 0.002087 | 0.048377 |
| 36 | Creatine | 261.1297 | 327.69 | 1.490305 | 1.64E-06 | 2.920886 |
| 37 | Creatinine | 112.0508 | 160.192 | 1.448833 | 0.016 | 8.717168 |
| 38 | Cyclohexylsulfamate | 238.0733 | 81.1885 | 1.50998 | 0.000581 | 16.0586 |
| 39 | Cysteine-S-sulfate | 199.9675 | 279.587 | 1.431834 | 7.1E-05 | 0.481083 |
| 40 | Cytidine monophosphate N-acetylneuraminic acid | 613.1331 | 406.897 | 1.121163 | 0.015929 | 1.752394 |
| 41 | Cytosine | 147.9883 | 117.949 | 1.443571 | 8.06E-07 | 0.284192 |
| 42 | D(-)-beta-hydroxy butyric acid | 103.0392 | 185.604 | 1.424273 | 0.007806 | 3.238782 |
| 43 | D-Arabinono-1,4-lactone | 207.0493 | 74 | 1.404565 | 0.00147 | 1.779814 |
| 44 | D-Biotin | 243.0808 | 367.156 | 1.138573 | 0.00289 | 1.748328 |
| 45 | Deoxycholic acid | 391.2813 | 145.103 | 1.174237 | 0.005749 | 0.330839 |
| 46 | Deoxyinosine | 251.0763 | 170.04 | 1.229718 | 0.001113 | 1.642425 |
| 47 | Dexpanthenol | 221.1526 | 46.1875 | 1.374293 | 0.000456 | 2.113268 |
| 48 | D-galacturonic acid | 193.0337 | 364.266 | 1.073004 | 0.013551 | 0.640662 |
| 49 | Dihomo-gamma-Linolenic Acid | 305.2448 | 41.642 | 1.220782 | 0.001411 | 0.774426 |
| 50 | Dihydroxyacetone | 71.0134 | 373.899 | 1.298204 | 0.000483 | 0.689755 |
| 51 | DL-2-Aminoadipic acid | 160.0606 | 379.8 | 1.488242 | 0.001325 | 2.873443 |
| 52 | DL-2-Phosphoglycerate | 184.984 | 487.051 | 1.185978 | 0.000287 | 0.435686 |
| 53 | DL-lactate | 89.02469 | 216.599 | 1.378657 | 0.000104 | 0.485097 |
| 54 | Dodecanoic acid | 199.1689 | 46.662 | 1.176137 | 0.006348 | 1.360937 |
| 55 | D-Quinovose | 223.0804 | 196.2325 | 1.316566 | 0.000194 | 0.660944 |
| 56 | D-Ribulose 5-phosphate | 210.9992 | 269.402 | 1.021518 | 0.016104 | 1.522799 |
| 57 | EDTA | 291.0805 | 484.365 | 1.074282 | 0.003534 | 0.35983 |
| 58 | Ellipticine | 245.1124 | 287.425 | 1.452652 | 0.000179 | 0.112584 |
| 59 | Erucic acid | 337.3075 | 39.794 | 1.241075 | 0.007625 | 2.042697 |
| 60 | Floxuridine | 246.0601 | 23.438 | 1.19865 | 0.025136 | 3.011338 |
| 61 | Fludrocortisone acetate | 459.158 | 165.4745 | 1.166311 | 0.00808 | 2.023972 |
| 62 | Fosfomycin | 197.0205 | 367.068 | 1.270707 | 0.000872 | 1.76598 |
| 63 | Galactonic acid | 195.0498 | 350.3625 | 1.310245 | 6.91E-05 | 0.455377 |
| 64 | Glucosaminic acid | 254.0859 | 373.2515 | 1.14112 | 0.004496 | 0.682154 |
| 65 | Glucotropaeolin | 430.0349 | 396.156 | 1.381954 | 0.000132 | 2.315404 |
| 66 | Glycine | 74.02428 | 260.5945 | 1.176663 | 0.001914 | 1.575832 |
| 67 | Glycocholic acid | 464.3095 | 184.609 | 1.507847 | 6.73E-05 | 6.339164 |
| 68 | Glycyl-L-leucine | 187.1074 | 265.0145 | 1.422446 | 2.49E-05 | 0.413513 |
| 69 | Guanidoacetic acid | 116.0455 | 332.755 | 1.357352 | 5.79E-06 | 0.256997 |
| 70 | Guanosine | 282.0826 | 246.41 | 1.324329 | 0.001802 | 0.489167 |
| 71 | Hesperetin | 318.0927 | 367.247 | 1.242465 | 0.000902 | 1.686585 |
| 72 | Hexanoylglycine | 172.0971 | 162.086 | 1.322305 | 0.001403 | 2.605242 |
| 73 | Hydroxyacetone | 133.0496 | 266.904 | 1.278564 | 0.000911 | 0.65376 |
| 74 | Hypotaurine | 108.0115 | 323.58 | 1.224671 | 0.001028 | 0.376079 |
| 75 | Hypoxanthine | 135.0302 | 204.61 | 1.513677 | 9.68E-08 | 0.51382 |
| 76 | Immunomycin | 791.4801 | 39.762 | 1.483772 | 0.00038 | 3.345997 |
| 77 | Indole-3-carboxylic acid | 160.0394 | 38.525 | 1.225429 | 0.000491 | 4.2635 |
| 78 | Indoxyl sulfate | 213.0108 | 25.559 | 1.523736 | 0.000295 | 11.06318 |
| 79 | Inosine | 267.0703 | 266.737 | 1.481506 | 5.8E-06 | 0.55788 |
| 80 | Isobutyrylglycine | 204.0842 | 239.933 | 1.230959 | 0.002489 | 0.777514 |
| 81 | Isopimaric acid | 301.2146 | 42.372 | 1.427042 | 0.000498 | 2.016805 |
| 82 | Isovalerylglycine | 158.0812 | 177.142 | 1.40393 | 2.36E-05 | 3.214252 |
| 83 | Isoxanthopterin | 215.9956 | 150.6275 | 1.300563 | 0.005432 | 3.850343 |
| 84 | Jasmonic acid | 226.1428 | 124.878 | 1.342354 | 3.86E-05 | 0.53596 |
| 85 | Kainic Acid | 234.0775 | 44.29 | 1.400127 | 0.002065 | 11.43602 |
| 86 | Kynurenic acid | 188.0341 | 166.2225 | 1.498195 | 0.000136 | 16.60212 |
| 87 | L-Alanine | 88.04022 | 327.475 | 1.250354 | 0.001388 | 1.38112 |
| 88 | L-Arabitol | 189.0947 | 204.55 | 1.386487 | 0.006536 | 7.16283 |
| 89 | L-Aspartate | 132.0291 | 473.54 | 1.220892 | 0.001498 | 0.645147 |
| 90 | L-Carnitine | 220.1171 | 339.115 | 1.456068 | 1.1E-05 | 0.55955 |
| 91 | L-Citrulline | 174.0875 | 371.2735 | 1.135561 | 0.008831 | 0.54877 |
| 92 | L-Cystine | 239.0141 | 474.27 | 1.174047 | 0.000979 | 0.555825 |
| 93 | L-Glutamate | 146.0459 | 368.639 | 1.179048 | 0.002344 | 0.740924 |
| 94 | L-Glutamine | 145.0616 | 351.624 | 1.006291 | 0.015577 | 0.789918 |
| 95 | Linoleic acid | 279.23 | 102.7305 | 1.50299 | 0.000328 | 2.884154 |
| 96 | L-Isoleucine | 130.0867 | 268.889 | 1.244536 | 0.007574 | 0.708617 |
| 97 | L-Leucine | 130.0869 | 245.549 | 1.153439 | 0.009448 | 0.77571 |
| 98 | L-Methionine | 148.0424 | 179.57 | 1.465877 | 4.61E-06 | 0.390394 |
| 99 | L-Phenylalanine | 164.0711 | 238.468 | 1.072015 | 0.029872 | 0.782192 |
| 100 | L-Pipecolic acid | 128.0705 | 290.008 | 1.379533 | 0.017397 | 2.533125 |
| 101 | L-Proline | 114.0556 | 292.095 | 1.335116 | 0.005512 | 0.578278 |
| 102 | L-Saccharopine | 275.1221 | 419.384 | 1.497614 | 0.000624 | 3.035481 |
| 103 | L-Serine | 104.0345 | 356.399 | 1.270633 | 0.000353 | 0.579946 |
| 104 | L-Tryptophan | 203.0811 | 239.933 | 1.217179 | 0.003734 | 0.775323 |
| 105 | L-Tyrosine | 180.0654 | 281.218 | 1.441101 | 1.53E-05 | 0.530875 |
| 106 | Lumichrome | 241.0714 | 50.86 | 1.407801 | 2.35E-05 | 0.353036 |
| 107 | Maleamic acid | 114.0186 | 355.388 | 1.191752 | 0.001805 | 0.672327 |
| 108 | Maleic acid | 175.0237 | 414.865 | 1.081342 | 0.005746 | 0.351767 |
| 109 | Malonic acid | 103.0028 | 375.64 | 1.40833 | 0.012073 | 3.263695 |
| 110 | Methylthiouracil | 158.0387 | 172.6745 | 1.519735 | 0.00236 | 9.79718 |
| 111 | Muramic acid | 288.053 | 31.302 | 1.493793 | 0.000253 | 12.3856 |
| 112 | Myristic acid | 287.2192 | 45.583 | 1.29297 | 0.005689 | 2.31525 |
| 113 | Myristoleic acid | 225.1843 | 44.929 | 1.282013 | 0.005297 | 2.143592 |
| 114 | N-.alpha.-Acetyl-L-arginine | 215.1099 | 180.023 | 1.342088 | 0.000518 | 1.838893 |
| 115 | N1-Methyl-2-pyridone-5-carboxamide | 151.0501 | 82.639 | 1.389163 | 0.006901 | 11.4126 |
| 116 | N2-Acetyl-L-ornithine | 211.0455 | 126.235 | 1.498355 | 0.000417 | 3.723499 |
| 117 | N4-Acetylcytidine | 284.0826 | 204.384 | 1.449374 | 1.16E-06 | 3.219816 |
| 118 | N6-Acetyl-L-lysine | 187.1073 | 328.705 | 1.307333 | 0.000625 | 0.454379 |
| 119 | N-Acetylaspartate | 191.066 | 327.647 | 1.426767 | 3.45E-05 | 0.357895 |
| 120 | N-Acetyl-DL-methionine | 190.0531 | 179.538 | 1.474501 | 3.2E-06 | 0.31983 |
| 121 | N-Acetyl-L-alanine | 130.0499 | 233.648 | 1.361011 | 0.000124 | 0.476437 |
| 122 | N-Acetyl-L-aspartic acid | 196.0268 | 159.654 | 1.219827 | 0.003982 | 1.566466 |
| 123 | N-Acetylmannosamine | 202.0701 | 143.551 | 1.500844 | 9.52E-08 | 2.123205 |
| 124 | N-Carboxyethyl-.gamma.-aminobutyric acid | 212.038 | 175.559 | 1.472212 | 5.13E-06 | 3.228547 |
| 125 | Nervonic acid | 365.3384 | 39.181 | 1.24781 | 0.018137 | 3.473363 |
| 126 | Nicotinamide | 121.0398 | 62.014 | 1.491637 | 5.13E-07 | 0.508246 |
| 127 | Nicotinamide adenine dinucleotide (NAD) | 662.1019 | 411.5835 | 1.145866 | 0.005287 | 0.502799 |
| 128 | Nomilin | 551.1585 | 204.47 | 1.397985 | 0.000212 | 17.91611 |
| 129 | O-Phospho-L-homoserine | 198.0156 | 383.1 | 1.484824 | 5.75E-06 | 1.807539 |
| 130 | O-Succinyl-L-homoserine | 256.0262 | 34.511 | 1.450001 | 5.47E-06 | 4.461928 |
| 131 | Oxazepam | 267.0326 | 291.2645 | 1.02183 | 0.024292 | 1.608072 |
| 132 | Pantothenate | 218.1021 | 251.193 | 1.354259 | 0.004327 | 0.53183 |
| 133 | p-Cresol | 107.0527 | 22.808 | 1.51877 | 4.41E-06 | 211.1005 |
| 134 | Pentadecanoic Acid | 241.2153 | 44.29 | 1.124122 | 0.013385 | 1.301594 |
| 135 | Perseitol | 193.0693 | 165.864 | 1.469636 | 0.000735 | 3.934217 |
| 136 | Phenethyl Caffeiate | 265.0808 | 329.786 | 1.038696 | 0.01887 | 0.773508 |
| 137 | Phenylacetylglycine | 192.0659 | 165.514 | 1.534177 | 0.000466 | 20.4378 |
| 138 | Phenylpropionylglycine | 228.0636 | 370.739 | 1.32574 | 3.81E-05 | 2.641719 |
| 139 | Phosphoenolpyruvate | 188.9609 | 417.7475 | 1.423305 | 6.48E-05 | 0.393667 |
| 140 | Phosphorylcholine | 365.1163 | 273.1445 | 1.166829 | 0.003483 | 1.502586 |
| 141 | Primidone | 218.1096 | 306.533 | 1.381337 | 0.009606 | 4.560461 |
| 142 | Ribitol | 151.06 | 223.318 | 1.336652 | 4.45E-05 | 0.217155 |
| 143 | Riboflavin | 375.1263 | 198.258 | 1.153731 | 0.005685 | 0.612976 |
| 144 | Rutin | 669.1598 | 246.305 | 1.244832 | 0.018496 | 0.420978 |
| 145 | Saccharin | 242.012 | 172.531 | 1.227683 | 0.000757 | 2.506546 |
| 146 | S-Adenosyl-L-homocysteine | 383.1176 | 281.341 | 1.422014 | 0.000786 | 0.366716 |
| 147 | Salicyluric acid | 194.0441 | 217.392 | 1.535804 | 8.79E-05 | 5.332261 |
| 148 | Salidroside | 321.092 | 152.013 | 1.074258 | 0.016015 | 0.686494 |
| 149 | S-Allyl-L-cysteine | 142.032 | 107.421 | 1.107637 | 0.012172 | 1.517492 |
| 150 | Sebacic acid | 201.1114 | 296.462 | 1.341661 | 0.013997 | 3.337078 |
| 151 | sn-Glycerol 1-phosphate | 188.0914 | 250.438 | 1.496786 | 9.46E-05 | 4.24678 |
| 152 | sn-Glycerol 3-phosphoethanolamine | 214.0478 | 373.045 | 1.067441 | 0.008327 | 1.488272 |
| 153 | Stavudine | 205.0637 | 271.332 | 1.291542 | 0.000812 | 0.414706 |
| 154 | Stearidonic Acid | 275.1989 | 43.646 | 1.205787 | 0.002782 | 1.52413 |
| 155 | T0901317 | 481.0351 | 369.272 | 1.020495 | 0.035246 | 0.800876 |
| 156 | Taurine | 124.0074 | 276.46 | 1.019245 | 0.029985 | 0.841837 |
| 157 | Tetracosanoic acid | 367.3541 | 39.709 | 1.122286 | 0.028717 | 3.562462 |
| 158 | Thiamine monophosphate | 365.0461 | 205.2115 | 1.247363 | 0.002293 | 0.815081 |
| 159 | Thymine | 125.0346 | 75.4855 | 1.016391 | 0.031857 | 1.445413 |
| 160 | Undecanoic Acid | 185.1532 | 46.845 | 1.077139 | 0.006851 | 0.695047 |
| 161 | Uracil mustard | 251.0205 | 52.363 | 1.504022 | 0.001968 | 5.924231 |
| 162 | Uridine | 303.0778 | 251.193 | 1.432883 | 2.96E-05 | 0.555732 |
| 163 | Urocanic acid | 174.992 | 22.955 | 1.51569 | 7.63E-05 | 29.27497 |
| 164 | Vanylglycol | 165.0552 | 39.385 | 1.270499 | 0.001813 | 1.919028 |
| 165 | Vindoline | 437.2091 | 251.2805 | 1.31986 | 0.011101 | 0.35557 |
| 166 | Xanthurenic acid | 204.0316 | 165.418 | 1.465592 | 6.55E-07 | 2.430977 |
| 167 | Zearalenone | 317.143 | 436.4355 | 1.188988 | 2.04E-05 | 0.288755 |

**Supplementary Table 2.** Significantly differentially expressed metabolites in the kidney of AKI mice *versus* control mice in positive ion mode

| NO. | Metabolites | m/z | Rt (min) | VIP | P Value | Fold Change |
| --- | --- | --- | --- | --- | --- | --- |
| 1 | (-)-Medicarpin | 293.0716 | 204.668 | 1.10878 | 0.015631 | 0.824189 |
| 2 | (-)-Riboflavin | 341.1206 | 46.772 | 1.212388 | 0.014893 | 0.494839 |
| 3 | (3-Carboxypropyl)trimethylammonium cation | 146.1164 | 357.6845 | 1.138149 | 0.00412 | 0.757355 |
| 4 | (4Z,7Z,10Z,13Z,16Z,19Z)-4,7,10,13,1 6,19-Docosahexaenoic acid | 311.2334 | 33.8885 | 1.34619 | 0.000122 | 1.886366 |
| 5 | (R)-(+)-Citronellic acid | 188.1619 | 183.4 | 1.295863 | 0.00733 | 0.345902 |
| 6 | (R)-3-Hydroxybutyric acid | 247.0549 | 320.134 | 1.346691 | 0.0007 | 1.563328 |
| 7 | .alpha.-L-Asp-L-Lys | 303.1631 | 417.011 | 1.185936 | 0.000191 | 0.486386 |
| 8 | .beta.-Cyano-L-alanine | 175.069 | 203.258 | 1.525631 | 0.002916 | 7.29703 |
| 9 | .beta.-Homoproline | 276.1887 | 383.867 | 1.417967 | 3.14E-05 | 1.572015 |
| 10 | .gamma.-L-Glu-.epsilon.-L-Lys | 317.1749 | 347.4075 | 1.224088 | 0.021729 | 1.828451 |
| 11 | 1-Aminocyclohexanecarboxylic acid | 144.0997 | 328.9995 | 1.309091 | 0.003407 | 0.450221 |
| 12 | 1-Methylnicotinamide | 137.0691 | 313.751 | 1.431714 | 0.000671 | 3.652045 |
| 13 | 1-Oleoyl-L-.alpha.-lysophosphatidic acid | 478.2873 | 182.7015 | 1.195316 | 0.004496 | 0.831079 |
| 14 | 1-O-Octadecyl-sn-glyceryl-3-phosphorylcholine | 509.3721 | 165.358 | 1.191804 | 0.003099 | 1.640208 |
| 15 | 1-Phenylethylamine | 144.0787 | 182.738 | 1.478791 | 6.14E-06 | 0.422642 |
| 16 | 1-Stearoyl-2-hydroxy-sn-glycero-3-phosphoethanolamine | 482.3171 | 156.862 | 1.334711 | 0.000948 | 0.627041 |
| 17 | 2(1H)-Pyridinone | 96.04295 | 61.957 | 1.486334 | 1.25E-05 | 0.595214 |
| 18 | 2-Butoxyethanol | 182.1151 | 42.027 | 1.188054 | 0.004109 | 0.572111 |
| 19 | 2'-Deoxyadenosine 5'-monophosphate (dAMP) | 376.0305 | 83.179 | 1.515643 | 0.006726 | 9.948563 |
| 20 | 2-Hydroxy-3-methoxybenzoic acid | 168.0413 | 275.959 | 1.252804 | 0.002829 | 0.683368 |
| 21 | 2-Hydroxypyridine | 78.03288 | 61.957 | 1.444463 | 5.82E-05 | 0.589779 |
| 22 | 2-Ketobutyric acid | 103.0373 | 343.939 | 1.381061 | 0.000155 | 0.69673 |
| 23 | 2-Methylbutyroylcarnitine | 246.1676 | 224.241 | 1.3327 | 0.006341 | 0.501557 |
| 24 | 2-Methylguanosine | 339.1368 | 168.635 | 1.451321 | 0.001399 | 3.515442 |
| 25 | 2'-O-methylcytidine | 324.0619 | 24.705 | 1.407398 | 0.002056 | 0.522065 |
| 26 | 2'-O-methylinosine | 324.1378 | 231.322 | 1.321715 | 0.000229 | 0.54711 |
| 27 | 2-Phenylacetamide | 288.1771 | 171.968 | 1.48535 | 0.002104 | 0.188774 |
| 28 | 3-Butynoic acid | 148.0406 | 182.073 | 1.365918 | 0.000513 | 0.351769 |
| 29 | 3-Furancarboxylic acid | 176.0351 | 424.602 | 1.457854 | 3.36E-05 | 1.898094 |
| 30 | 3-Hydroxyanthranilic acid | 154.0477 | 91.7425 | 1.508106 | 0.008209 | 12.802 |
| 31 | 3-Hydroxyisovaleric acid | 182.0785 | 417.044 | 1.465051 | 2.28E-06 | 0.347785 |
| 32 | 3-Ureidopropionate | 133.0588 | 294.019 | 1.170241 | 0.023739 | 0.558622 |
| 33 | 4-acetamidobutanoate | 206.1009 | 308.0955 | 1.451345 | 0.000387 | 0.231829 |
| 34 | 4-Guanidinobutyric acid | 146.0902 | 338.735 | 1.337006 | 0.000597 | 2.744286 |
| 35 | 4-Pyridoxic acid | 184.0582 | 39.2805 | 1.548799 | 0.00014 | 26.29021 |
| 36 | 5,2'-O-dimethyluridine | 583.1672 | 434.101 | 1.406143 | 8.88E-05 | 0.489745 |
| 37 | 5'-Deoxyadenosine | 293.139 | 22.9205 | 1.377707 | 0.000242 | 1.721749 |
| 38 | 5-Hydroxymethylcytidine | 256.0918 | 395.5475 | 1.308009 | 0.000205 | 2.001775 |
| 39 | 5-L-Glutamyl-L-alanine | 219.0948 | 379.963 | 1.341517 | 0.000562 | 0.701461 |
| 40 | 5-methoxyuridine | 274.0838 | 158.555 | 1.387269 | 4.39E-05 | 0.40345 |
| 41 | 5-Methyl-5,6-Dihydrouracil | 146.0902 | 304.31 | 1.455607 | 0.014521 | 8.596366 |
| 42 | 5-Methylcytidine | 258.1054 | 238.209 | 1.473098 | 0.000358 | 4.303322 |
| 43 | 5-Methylcytosine | 126.0644 | 238.227 | 1.465509 | 0.000179 | 4.553671 |
| 44 | 6-Benzylaminopurine | 267.1301 | 47.271 | 1.317746 | 0.002206 | 0.423866 |
| 45 | 7-Oxocholesterol | 401.3363 | 33.513 | 1.078614 | 0.027929 | 0.603179 |
| 46 | Acadesine (Drug) | 259.1006 | 186.1735 | 1.397154 | 0.005522 | 5.473966 |
| 47 | Acetic acid, phenyl ester | 137.0629 | 149.7195 | 1.429242 | 9.03E-05 | 0.301859 |
| 48 | Acetoacetic acid | 120.0637 | 68.281 | 1.167018 | 0.023942 | 0.435897 |
| 49 | Acetohydroxamic acid | 120.0015 | 361.758 | 1.138508 | 0.009487 | 0.723698 |
| 50 | Acetylcholine | 146.1154 | 187.627 | 1.53803 | 1.04E-05 | 0.295502 |
| 51 | Acetylcysteine | 164.0353 | 193.744 | 1.204308 | 0.032593 | 0.302188 |
| 52 | Acetyl-DL-Valine | 319.1907 | 347.067 | 1.52216 | 0.000766 | 5.519038 |
| 53 | Adenine | 136.0608 | 149.8 | 1.464444 | 4.01E-05 | 0.340414 |
| 54 | Ala-Gly | 207.0941 | 308.101 | 1.450006 | 0.000485 | 0.235764 |
| 55 | Allocystathionine | 264.1045 | 195.208 | 1.283848 | 0.034884 | 2.309275 |
| 56 | alpha-Guanidinoglutaric Acid | 228.0352 | 354.319 | 1.410034 | 0.000128 | 0.358613 |
| 57 | Anthranilic acid (Vitamin L1) | 137.0442 | 323.4485 | 1.019095 | 0.030146 | 0.760399 |
| 58 | Arachidonic Acid (peroxide free) | 368.2571 | 45.754 | 1.165025 | 0.003022 | 0.581848 |
| 59 | Arachidonoyl ethanolamide | 348.2856 | 35.384 | 1.535744 | 3.09E-07 | 0.395299 |
| 60 | Arg-Ala | 245.154 | 233.957 | 1.379666 | 0.005585 | 0.439319 |
| 61 | Arg-Cys | 319.1576 | 437.462 | 1.275299 | 0.001001 | 0.409366 |
| 62 | Arg-Glu | 304.1588 | 425.484 | 1.379395 | 0.000236 | 0.652586 |
| 63 | Argininosuccinic acid | 291.127 | 438.617 | 1.081207 | 0.015767 | 1.213765 |
| 64 | Arg-Thr | 276.1631 | 403.702 | 1.312384 | 0.000795 | 0.545012 |
| 65 | Asn-Pro | 252.0958 | 98.568 | 1.485118 | 9.75E-06 | 0.280384 |
| 66 | Azacitidine | 267.0693 | 148.7965 | 1.192483 | 0.006969 | 0.538707 |
| 67 | Baclofen | 255.0925 | 289.052 | 1.332618 | 0.019301 | 3.998477 |
| 68 | Betaine | 118.0855 | 256.156 | 1.49845 | 0.000174 | 0.543793 |
| 69 | Betaine aldehyde | 102.0902 | 389.79 | 1.0626 | 0.031236 | 0.669631 |
| 70 | beta-Nicotinamide D-ribonucleotide | 335.0598 | 476.251 | 1.066849 | 0.013893 | 0.485788 |
| 71 | Bethanechol cation | 238.0425 | 372.842 | 1.19691 | 0.002104 | 1.542882 |
| 72 | Butoxyacetic acid | 132.0789 | 141.632 | 1.51566 | 3.52E-08 | 0.320309 |
| 73 | Cer(d18:1/18:1(9Z)) | 546.5167 | 33.939 | 1.213796 | 0.023588 | 4.985893 |
| 74 | Cholic acid | 426.3159 | 206.482 | 1.377341 | 0.009684 | 0.132051 |
| 75 | Codeine | 317.1893 | 419.382 | 1.350166 | 0.000391 | 0.600853 |
| 76 | Corticosterone | 347.2177 | 40.983 | 1.403924 | 2.69E-05 | 2.879084 |
| 77 | Coumarin | 147.0418 | 281.2905 | 1.457588 | 0.00045 | 0.563349 |
| 78 | Creatine | 285.1245 | 327.761 | 1.394074 | 0.000925 | 1.450118 |
| 79 | Creatinine | 114.0649 | 159.392 | 1.557919 | 7.22E-12 | 4.313492 |
| 80 | Crotonic acid | 190.1046 | 204.709 | 1.461847 | 0.003077 | 8.955327 |
| 81 | Cyclohexylamine | 141.1364 | 194.842 | 1.14826 | 0.008719 | 1.345998 |
| 82 | Cysteine-S-sulfate | 201.981 | 280.731 | 1.115066 | 0.019916 | 0.64686 |
| 83 | Cytidine 2',3'-cyclic phosphate | 306.0452 | 296.648 | 1.478414 | 0.002679 | 3.035103 |
| 84 | Cytidine 5'-diphosphocholine (CDP-choline) | 489.109 | 415.84 | 1.01215 | 0.029527 | 1.626815 |
| 85 | Cytidine 5'-monophosphate | 324.0553 | 419.63 | 1.395304 | 3.12E-05 | 1.842373 |
| 86 | Cytidine 5'-monophosphate (CMP) | 346.037 | 419.771 | 1.470204 | 1.27E-05 | 1.817631 |
| 87 | Cytidine monophosphate N-acetylneuraminic acid | 653.1231 | 421.099 | 1.499494 | 0.00028 | 1.798228 |
| 88 | Daidzein | 255.0617 | 46.3355 | 1.029694 | 0.033907 | 0.425998 |
| 89 | D-Aspartic acid | 134.0427 | 375.627 | 1.427243 | 0.000105 | 0.611114 |
| 90 | D-Biotin | 262.1258 | 372.148 | 1.312343 | 0.001275 | 0.504928 |
| 91 | d-Dethiobiotin | 197.1259 | 299.9505 | 1.360056 | 7.98E-05 | 0.580822 |
| 92 | Decanoyl-L-carnitine | 316.2445 | 174.48 | 1.111609 | 0.01162 | 0.531733 |
| 93 | Deoxycytidine | 228.0953 | 193.789 | 1.331811 | 0.000939 | 3.314649 |
| 94 | Deoxyinosine | 294.1162 | 83.963 | 1.49716 | 0.001703 | 0.31914 |
| 95 | Desoxypeganine | 362.2319 | 36.854 | 1.301816 | 0.007809 | 2.383064 |
| 96 | D-Glucuronate | 159.0258 | 204.513 | 1.361142 | 0.000529 | 0.766049 |
| 97 | Diethanolamine | 70.06488 | 292.556 | 1.110229 | 0.039835 | 0.79797 |
| 98 | Dihydro-4,4-dimethyl-2,3-furandione | 189.0746 | 151.901 | 1.46598 | 0.001332 | 0.43314 |
| 99 | DL-2-Aminoadipic acid | 206.0374 | 380.4435 | 1.468786 | 0.001969 | 3.178727 |
| 100 | DL-Homocysteine | 118.0303 | 289.951 | 1.342409 | 0.000148 | 0.274241 |
| 101 | DL-Homoserine | 161.09 | 98.884 | 1.327362 | 0.001706 | 0.375171 |
| 102 | DL-Indole-3-lactic acid | 188.0688 | 240.118 | 1.209123 | 0.003954 | 0.711323 |
| 103 | DL-Methionine sulfoxide | 166.0555 | 281.353 | 1.37256 | 0.000507 | 0.609296 |
| 104 | Donepezil | 418.1708 | 34.159 | 1.250169 | 0.002243 | 2.080365 |
| 105 | Dopamine | 214.1046 | 198.479 | 1.449925 | 0.000806 | 3.152612 |
| 106 | D-Pipecolinic acid | 130.0845 | 290.244 | 1.374728 | 0.0406 | 3.39817 |
| 107 | Duloxetine | 262.1076 | 87.7595 | 1.507924 | 0.000966 | 7.395196 |
| 108 | Ethyl glucuronide | 240.1076 | 522.947 | 1.118916 | 0.004792 | 1.803791 |
| 109 | Fexofenadine | 1003.563 | 180.115 | 1.357857 | 0.013081 | 0.472433 |
| 110 | Flavin adenine dinucleotide (FAD) | 786.155 | 367.148 | 1.499582 | 1.66E-05 | 0.551271 |
| 111 | Formylanthranilic acid | 148.0405 | 309.984 | 1.342661 | 0.000567 | 0.750707 |
| 112 | gamma-L-Glutamyl-L-glutamic acid | 277.1003 | 426.392 | 1.2329 | 0.002575 | 1.945258 |
| 113 | Gln-Asn | 225.0969 | 370.896 | 1.197328 | 0.002155 | 1.387154 |
| 114 | Glycogen | 689.2027 | 474.67 | 1.233019 | 0.00468 | 1.778141 |
| 115 | Glycyl-L-leucine | 227.0846 | 370.283 | 1.108456 | 0.015081 | 1.54905 |
| 116 | Glycylproline | 155.0794 | 324.464 | 1.388739 | 0.02279 | 5.127117 |
| 117 | Gly-Trp | 262.12 | 324.631 | 1.432617 | 0.000939 | 0.580076 |
| 118 | Guanidine | 120.0789 | 38.1935 | 1.347523 | 0.000581 | 0.637995 |
| 119 | Guanidinosuccinic acid | 176.0644 | 375.496 | 1.32501 | 0.018399 | 3.19453 |
| 120 | Guanidoacetic acid | 118.0633 | 239.749 | 1.098003 | 0.016255 | 0.898572 |
| 121 | Guanosine | 284.0967 | 246.584 | 1.375145 | 0.005697 | 0.521198 |
| 122 | Hexanoylglycine | 174.11 | 162.167 | 1.292953 | 0.001791 | 2.090338 |
| 123 | His-Pro | 235.1158 | 134.558 | 1.459164 | 0.000807 | 2.748384 |
| 124 | His-Thr | 257.1211 | 320.324 | 1.384272 | 0.001345 | 0.499123 |
| 125 | Homocitrate | 270.0611 | 370.266 | 1.040852 | 0.04313 | 2.022139 |
| 126 | Homovanillic acid | 382.1422 | 204.837 | 1.50807 | 3.31E-07 | 1.969868 |
| 127 | Hydrocortisone | 345.2018 | 39.263 | 1.402294 | 0.00567 | 3.023826 |
| 128 | Hypotaurine | 110.0252 | 323.744 | 1.293969 | 0.000766 | 0.391544 |
| 129 | Hypoxanthine | 137.0446 | 158.752 | 1.362139 | 0.000131 | 0.567208 |
| 130 | Ile-Ala | 279.0473 | 349.384 | 1.344941 | 0.000399 | 2.436623 |
| 131 | Ile-Asn | 284.1034 | 369.022 | 1.085421 | 0.025883 | 1.214705 |
| 132 | Ile-Cys | 276.141 | 247.954 | 1.19837 | 0.001878 | 0.48657 |
| 133 | Ile-Met | 542.3157 | 175.73 | 1.19555 | 0.005971 | 0.661731 |
| 134 | Ile-Ser | 260.1574 | 456.657 | 1.45645 | 3.57E-06 | 0.411013 |
| 135 | Ile-Thr | 274.1731 | 462.479 | 1.471218 | 2.83E-06 | 0.500626 |
| 136 | Imidazole | 110.0695 | 291.1945 | 1.374114 | 0.009196 | 2.845384 |
| 137 | Indole-2-carboxylic acid | 144.0454 | 43.812 | 1.408433 | 8.38E-05 | 0.352834 |
| 138 | Inosine | 269.0858 | 204.556 | 1.522428 | 6.1E-07 | 0.577183 |
| 139 | Isobutyrylglycine | 146.0788 | 197.5755 | 1.080832 | 0.009268 | 1.223551 |
| 140 | Isovaleric acid | 120.1002 | 328.229 | 1.431871 | 0.002382 | 0.358248 |
| 141 | Jasmine lactone | 354.2597 | 42.151 | 1.41544 | 4.26E-05 | 1.848817 |
| 142 | Kynurenic acid | 250.0676 | 371.894 | 1.290923 | 0.002261 | 1.342726 |
| 143 | L-Alanine | 90.05411 | 327.759 | 1.376675 | 0.000153 | 1.277934 |
| 144 | L-Carnitine | 162.1109 | 372.1925 | 1.530978 | 6.37E-07 | 0.480676 |
| 145 | L-Cysteine | 122.0254 | 193.264 | 1.288066 | 0.012215 | 0.330465 |
| 146 | Leu-Ala | 203.1365 | 44.898 | 1.362959 | 0.001377 | 0.448782 |
| 147 | Leu-Leu | 245.1831 | 38.7595 | 1.418888 | 0.000151 | 0.333675 |
| 148 | Leu-Trp | 378.2075 | 388.978 | 1.407141 | 2.75E-05 | 0.418249 |
| 149 | Leu-Val | 231.1674 | 356.717 | 1.512654 | 6.2E-07 | 2.242761 |
| 150 | Linalool oxide | 231.157 | 282.098 | 1.481227 | 0.010779 | 4.560085 |
| 151 | Linoleic acid | 344.258 | 46.29 | 1.36702 | 0.000229 | 0.362577 |
| 152 | Linoleoyl ethanolamide | 324.2862 | 35.754 | 1.50843 | 4.15E-06 | 0.51019 |
| 153 | L-Methionine | 150.056 | 264.4965 | 1.357032 | 0.000377 | 0.612165 |
| 154 | L-Proline | 116.0695 | 292.531 | 1.272874 | 0.007427 | 0.830895 |
| 155 | L-Pyroglutamic acid | 190.0686 | 479.1495 | 1.420745 | 0.002213 | 0.50888 |
| 156 | L-Saccharopine | 277.1367 | 420.361 | 1.508312 | 0.000294 | 3.2657 |
| 157 | L-Serine | 106.0482 | 355.8695 | 1.360784 | 0.00035 | 0.603576 |
| 158 | L-Threonate | 136.0378 | 91.265 | 1.4946 | 0.012811 | 39.27647 |
| 159 | L-Threonine | 84.0432 | 395.792 | 1.234801 | 0.002128 | 0.585311 |
| 160 | Lumichrome | 243.0858 | 51.036 | 1.520594 | 7.34E-08 | 0.397608 |
| 161 | L-Valine | 118.0843 | 281.342 | 1.476083 | 1.63E-05 | 0.723598 |
| 162 | Lys-Asp | 262.1445 | 387.132 | 1.050934 | 0.023169 | 1.2227 |
| 163 | Lys-Cys | 214.1048 | 421.024 | 1.506194 | 0.002076 | 3.059376 |
| 164 | Lys-His | 328.1393 | 205.349 | 1.416691 | 0.000206 | 0.719099 |
| 165 | Lys-Leu | 304.1583 | 372.847 | 1.473998 | 1.47E-06 | 0.418606 |
| 166 | Lys-Phe | 316.1577 | 470.7 | 1.484889 | 6.26E-06 | 0.559581 |
| 167 | Lys-Thr | 248.1574 | 420.581 | 1.206972 | 0.004077 | 0.73693 |
| 168 | Lys-Trp | 333.1977 | 428.936 | 1.079436 | 0.019172 | 0.64752 |
| 169 | Lys-Val | 228.1679 | 321.65 | 1.308981 | 0.025066 | 4.428614 |
| 170 | Mandelonitrile | 284.1434 | 234.8605 | 1.129328 | 0.02353 | 1.703919 |
| 171 | Met-Ala | 203.0821 | 322.3315 | 1.435537 | 0.002237 | 0.150221 |
| 172 | Methoprene (S) | 311.2543 | 39.5655 | 1.52209 | 0.000217 | 4.295735 |
| 173 | Methoxyacetic acid | 151.0593 | 143.7 | 1.557813 | 5.28E-07 | 5.462663 |
| 174 | Methyl acetoacetate | 134.0793 | 327.761 | 1.380708 | 0.000175 | 1.406461 |
| 175 | Mevalonic acid | 314.1785 | 425.11 | 1.45889 | 2.77E-05 | 0.495149 |
| 176 | MG(18:2(9Z,12Z)/0:0/0:0)[rac] | 355.2798 | 34.163 | 1.442464 | 6.09E-07 | 3.169144 |
| 177 | Molsidomine | 261.1418 | 433.9905 | 1.370405 | 5.11E-05 | 0.353508 |
| 178 | Monomethyl glutaric acid | 210.0712 | 226.15 | 1.484798 | 0.00319 | 8.435586 |
| 179 | N-.alpha.-Acetyl-L-arginine | 217.1267 | 350.654 | 1.119393 | 0.010762 | 1.32037 |
| 180 | N.alpha.-Acetyl-L-lysine | 189.1208 | 370.274 | 1.453321 | 0.002019 | 0.505171 |
| 181 | N1-Methyl-2-pyridone-5-carboxamide | 153.0648 | 87.7445 | 1.525755 | 0.005616 | 25.90009 |
| 182 | N4-Acetylcytidine | 286.0998 | 155.057 | 1.503582 | 1.09E-06 | 2.618539 |
| 183 | N6,N6,N6-Trimethyl-L-lysine | 189.1576 | 518.096 | 1.101881 | 0.013107 | 1.285326 |
| 184 | N6-Methyladenine | 337.1034 | 107.268 | 1.380568 | 0.00013 | 0.389765 |
| 185 | N-Acetyl-D-galactosamine | 336.0838 | 396.011 | 1.185017 | 0.001727 | 1.928067 |
| 186 | N-Acetyl-D-glucosamine | 222.0942 | 243.171 | 1.020007 | 0.017607 | 0.658501 |
| 187 | N-Acetyl-D-Glucosamine 6-Phosphate | 302.06 | 429.261 | 1.302672 | 0.000624 | 1.433332 |
| 188 | N-Acetyl-D-lactosamine | 384.1451 | 349.865 | 1.492062 | 0.001268 | 2.062178 |
| 189 | N-Acetylglutamine | 230.1189 | 402.275 | 1.331052 | 0.001524 | 1.409483 |
| 190 | N-Acetyl-L-glutamate | 172.0581 | 287.251 | 1.158437 | 0.00873 | 1.800884 |
| 191 | N-Acetyl-L-methionine | 192.0663 | 180.471 | 1.391156 | 0.00028 | 0.554697 |
| 192 | N-Acetylmannosamine | 186.0736 | 107.6485 | 1.094769 | 0.029608 | 0.600589 |
| 193 | N-Acetylserotonin | 219.1102 | 41.3245 | 1.011926 | 0.028383 | 0.657064 |
| 194 | Nadolol | 310.1977 | 185.3285 | 1.214871 | 0.008027 | 1.744131 |
| 195 | NG,NG-dimethyl-L-arginine(ADMA) | 203.1479 | 487.901 | 1.261855 | 0.001859 | 0.733507 |
| 196 | N-Glycolylneuraminic acid | 326.104 | 375.637 | 1.404865 | 0.005513 | 2.430475 |
| 197 | Nicotinic acid adenine dinucleotide (NAAD) | 664.1075 | 414.11 | 1.3469 | 0.001676 | 0.467019 |
| 198 | Nicotinuric acid | 378.1341 | 315.51 | 1.277152 | 0.002944 | 1.844063 |
| 199 | Nitrobenzene | 264.1008 | 25.13 | 1.549296 | 0.000615 | 28.02042 |
| 200 | N-Lignoceroylsphingosine | 632.6218 | 34.041 | 1.085333 | 0.014608 | 2.55593 |
| 201 | Nname,Cinoxacin | 263.0693 | 25.2805 | 1.522581 | 2.18E-05 | 8.817876 |
| 202 | Norharmane | 169.0734 | 44.263 | 1.155125 | 0.011352 | 0.473373 |
| 203 | N-Tigloylglycine | 158.0791 | 182.0855 | 1.448443 | 3.93E-05 | 2.01752 |
| 204 | O-Acetyl-L-serine | 148.0569 | 42.173 | 1.51345 | 0.000391 | 2.868752 |
| 205 | Oxyquinoline | 146.0608 | 180.1255 | 1.399574 | 0.005786 | 0.319017 |
| 206 | p-Acetamidophenol (Acetaminophen, Tylenol) | 320.1605 | 437.629 | 1.366431 | 7.42E-05 | 0.457029 |
| 207 | Palmitoyl ethanolamide | 360.3074 | 35.579 | 1.270839 | 0.033131 | 0.227157 |
| 208 | Pantothenate | 220.1158 | 251.375 | 1.326086 | 0.004755 | 0.520492 |
| 209 | Phe-Ala | 237.1201 | 40.273 | 1.103175 | 0.011504 | 0.502356 |
| 210 | Phenelzine | 159.0896 | 240.341 | 1.226525 | 0.002318 | 0.773535 |
| 211 | Phenylacetic acid | 119.0473 | 281.342 | 1.454352 | 0.000703 | 0.574415 |
| 212 | Phenylacetylglycine | 194.0786 | 166.019 | 1.531617 | 0.000755 | 18.745 |
| 213 | Phenyllactic acid | 131.0472 | 238.758 | 1.110877 | 0.012825 | 0.734989 |
| 214 | Phloretin | 335.1061 | 240.846 | 1.282721 | 0.002255 | 0.563909 |
| 215 | Phytosphingosine | 318.2965 | 146.0535 | 1.219587 | 0.026402 | 2.302517 |
| 216 | Pimelic acid | 202.1051 | 251.114 | 1.269193 | 0.011075 | 0.59983 |
| 217 | Primaquine | 336.0944 | 323.09 | 1.308654 | 0.038831 | 2.882582 |
| 218 | Pro-Ala | 187.1052 | 316.057 | 1.48451 | 0.000424 | 0.331492 |
| 219 | Pro-Asn | 271.1371 | 297.18 | 1.423372 | 0.013046 | 0.14102 |
| 220 | Pro-Glu | 245.1101 | 386.728 | 1.445846 | 0.001964 | 0.496722 |
| 221 | Promethazine | 361.0548 | 369.021 | 1.310216 | 0.000636 | 0.704474 |
| 222 | Propazine | 293.1187 | 297.112 | 1.397297 | 0.007037 | 0.257667 |
| 223 | Propoxur | 227.1442 | 273.749 | 1.214253 | 0.009849 | 2.164678 |
| 224 | Pro-Ser | 266.1117 | 355.086 | 1.456443 | 1.46E-05 | 0.473178 |
| 225 | Pro-Thr | 258.1472 | 256.104 | 1.490179 | 0.000202 | 0.409137 |
| 226 | Pro-Val | 275.1575 | 351.853 | 1.328134 | 0.000194 | 0.407248 |
| 227 | Pyridoxal (Vitamin B6) | 168.0631 | 98.265 | 1.514073 | 4.73E-06 | 0.379484 |
| 228 | Pyridoxal 5'-phosphate | 265.0527 | 482.8155 | 1.269459 | 0.00241 | 0.514971 |
| 229 | Pyridoxamine 5'-phosphate | 248.0584 | 25.7945 | 1.530679 | 0.00183 | 13.74642 |
| 230 | Quinaldic acid | 191.079 | 45.578 | 1.440226 | 2.57E-05 | 0.440613 |
| 231 | Quinolinate | 231.0325 | 26.125 | 1.539936 | 0.000419 | 10.53334 |
| 232 | Riboflavin | 377.141 | 198.502 | 1.201784 | 0.00365 | 0.592128 |
| 233 | Salicylic acid | 121.0264 | 32.709 | 1.113924 | 0.01036 | 0.8483 |
| 234 | Ser-Arg | 262.1475 | 404.624 | 1.282087 | 0.001999 | 0.57168 |
| 235 | sn-Glycerol 1-phosphate | 190.105 | 250.725 | 1.532038 | 0.000366 | 4.534766 |
| 236 | sn-Glycerol 3-phosphoethanolamine | 216.0606 | 372.847 | 1.282233 | 0.000404 | 1.654327 |
| 237 | Sotalol | 290.1565 | 369.014 | 1.353678 | 0.000136 | 1.871956 |
| 238 | Stearic acid | 302.3017 | 56.141 | 1.103474 | 0.024092 | 2.108764 |
| 239 | Succinate | 119.0333 | 204.634 | 1.50468 | 1.89E-06 | 0.566262 |
| 240 | Taurine | 126.0211 | 276.38 | 1.285827 | 0.002056 | 0.800866 |
| 241 | Thiamine | 265.1087 | 355.097 | 1.486946 | 3.03E-06 | 0.480739 |
| 242 | Thioetheramide-PC | 758.5622 | 131.4835 | 1.18868 | 0.002561 | 2.074603 |
| 243 | Thr-Thr | 221.1187 | 251.363 | 1.235624 | 0.006936 | 0.579586 |
| 244 | Thymine | 127.0481 | 99.488 | 1.196239 | 0.002827 | 0.782136 |
| 245 | Tolazoline | 178.1314 | 184.195 | 1.069662 | 0.005689 | 0.388854 |
| 246 | trans-cinnamate | 149.0573 | 238.7185 | 1.08239 | 0.02082 | 0.810246 |
| 247 | Triethanolamine | 132.1001 | 313.609 | 1.152533 | 0.014943 | 1.751333 |
| 248 | Trigonelline | 138.053 | 269.588 | 1.44205 | 1.06E-05 | 0.22716 |
| 249 | Tyramine | 120.0802 | 238.741 | 1.097832 | 0.015534 | 0.730342 |
| 250 | Tyr-Asp | 296.0952 | 107.416 | 1.273737 | 0.03075 | 0.359132 |
| 251 | UDP-N-acetylglucosamine | 608.0819 | 398.545 | 1.076045 | 0.02748 | 2.011729 |
| 252 | Urea | 61.03931 | 102.515 | 1.439377 | 0.001113 | 1.813617 |
| 253 | Val-Asn | 485.2288 | 311.33 | 1.504515 | 4.98E-07 | 0.341726 |
| 254 | Val-Val | 217.1519 | 355.769 | 1.434681 | 0.001866 | 3.521747 |
| 255 | Xanthosine | 285.0797 | 287.337 | 1.204974 | 0.002697 | 0.731396 |
| 256 | Xanthurenic acid | 206.0452 | 165.38 | 1.520705 | 3.86E-07 | 2.635196 |

**Supplementary Table 3.** Significantly differentially expressed metabolites in the kidney of AKI+JPYSF mice *versus* AKI mice in negative ion mode

| NO. | Metabolites | m/z | Rt (min) | VIP | P Value | Fold Change |
| --- | --- | --- | --- | --- | --- | --- |
| 1 | (R)-mevalonic acid 5-Phosphate | 265.0837 | 124.3605 | 2.021524 | 0.005818 | 0.268909 |
| 2 | 1,2-Benzenedicarboxylic acid | 165.0179 | 123.486 | 1.962155 | 0.000168 | 0.323112 |
| 3 | 11-Keto-.beta.-boswellic acid | 469.3262 | 48.4455 | 2.215823 | 0.002645 | 13.80174 |
| 4 | 2-keto-D-Gluconic acid | 210.0588 | 68.921 | 1.678075 | 0.034499 | 0.647235 |
| 5 | 3-Aminopropanesulphonic Acid | 175.9832 | 72.633 | 1.124679 | 0.041169 | 1.37709 |
| 6 | 3-Guanidinopropanoate | 152.0428 | 327.939 | 1.343849 | 0.044716 | 0.863816 |
| 7 | 3-Hydroxydodecanoic acid | 197.1533 | 46.47 | 1.362319 | 0.029182 | 0.640216 |
| 8 | 4-Pyridoxic acid | 182.0445 | 39.163 | 2.124887 | 1.52E-05 | 0.244309 |
| 9 | 5,6,7,8-tetrahydro-2-Naphthoic Acid | 157.0685 | 181.982 | 1.828442 | 0.003711 | 0.455844 |
| 10 | 8-iso-Prostaglandin A2 | 333.2035 | 72.105 | 1.348607 | 0.029638 | 0.430862 |
| 11 | 9R,10S-EpOME | 295.225 | 46.226 | 1.687706 | 0.006465 | 0.721825 |
| 12 | Acetyl phosphate | 138.979 | 425.823 | 1.438601 | 0.027123 | 1.401644 |
| 13 | Acetylvalerenolic acid | 583.3348 | 169.338 | 1.604477 | 0.002796 | 0.51677 |
| 14 | Adenosine | 304.0501 | 204.686 | 1.521903 | 0.024012 | 1.334643 |
| 15 | all cis-(6,9,12)-Linolenic acid | 277.215 | 43.02 | 1.584953 | 0.009208 | 0.63655 |
| 16 | Arachidonic Acid (peroxide free) | 303.2298 | 102.776 | 1.548041 | 0.012502 | 0.719222 |
| 17 | beta-Nicotinamide D-ribonucleotide | 356.0337 | 25.774 | 1.563225 | 0.023495 | 0.354676 |
| 18 | BHT | 219.1739 | 32.414 | 1.344999 | 0.029387 | 0.816133 |
| 19 | cis-Aconitate | 190.0341 | 422.237 | 1.321891 | 0.049506 | 1.917955 |
| 20 | Cysteine-S-sulfate | 199.9675 | 279.587 | 1.249336 | 0.048372 | 1.333625 |
| 21 | Cytosine | 147.9883 | 117.949 | 1.214183 | 0.03567 | 1.561044 |
| 22 | D-Arabinono-1,4-lactone | 207.0493 | 74 | 1.412734 | 0.036613 | 0.761131 |
| 23 | D-Biotin | 243.0808 | 367.156 | 1.57863 | 0.008039 | 0.629705 |
| 24 | Dexpanthenol | 221.1526 | 46.1875 | 1.396545 | 0.021859 | 0.681952 |
| 25 | DL-2-Aminoadipic acid | 160.0606 | 379.8 | 1.51071 | 0.019421 | 0.572759 |
| 26 | D-Ribose 5-phosphate | 289.0299 | 476.212 | 1.471665 | 0.020304 | 1.358948 |
| 27 | Ellipticine | 245.1124 | 287.425 | 1.560013 | 0.039885 | 1.979043 |
| 28 | Fosfomycin | 197.0205 | 367.068 | 1.575236 | 0.010704 | 0.66149 |
| 29 | Glycine | 74.02428 | 260.5945 | 1.825099 | 0.00147 | 1.24764 |
| 30 | Guanosine | 282.0826 | 246.41 | 1.40207 | 0.039939 | 1.391952 |
| 31 | Hesperetin | 318.0927 | 367.247 | 1.620497 | 0.006607 | 0.6577 |
| 32 | Hydroxyacetone | 133.0496 | 266.904 | 1.545596 | 0.02255 | 1.435495 |
| 33 | Indole-3-carboxylic acid | 160.0394 | 38.525 | 2.139669 | 0.001118 | 0.156495 |
| 34 | Indoxyl sulfate | 213.0108 | 25.559 | 1.437793 | 0.029107 | 0.529842 |
| 35 | Isopimaric acid | 301.2146 | 42.372 | 1.457199 | 0.046199 | 0.813466 |
| 36 | Isovalerylglycine | 158.0812 | 177.142 | 1.691883 | 0.014795 | 0.61592 |
| 37 | Isoxanthopterin | 215.9956 | 150.6275 | 1.779991 | 0.010202 | 0.352752 |
| 38 | Jasmonic acid | 226.1428 | 124.878 | 1.861997 | 0.013758 | 0.606683 |
| 39 | Kynurenic acid | 188.0341 | 166.2225 | 2.11204 | 2.82E-05 | 0.259003 |
| 40 | L-Arabitol | 189.0947 | 204.55 | 1.630811 | 0.031387 | 0.435631 |
| 41 | L-Carnitine | 220.1171 | 339.115 | 1.485651 | 0.016344 | 1.23679 |
| 42 | Linoleic acid | 279.23 | 102.7305 | 1.388385 | 0.028788 | 0.719885 |
| 43 | L-Saccharopine | 275.1221 | 419.384 | 1.476479 | 0.015353 | 0.525142 |
| 44 | Monomethyl glutaric acid | 183.0096 | 23.965 | 1.584326 | 0.012982 | 1.296669 |
| 45 | Muramic acid | 288.053 | 31.302 | 1.756417 | 0.002337 | 0.365285 |
| 46 | Myristoleic acid | 225.1843 | 44.929 | 1.2517 | 0.048681 | 0.692516 |
| 47 | N-Acetyl-DL-methionine | 190.0531 | 179.538 | 1.261464 | 0.044305 | 1.806572 |
| 48 | N-Carboxyethyl-.gamma.-aminobutyric acid | 212.038 | 175.559 | 1.713751 | 0.009382 | 0.68199 |
| 49 | Nicolsamide | 385.0059 | 155.805 | 1.481481 | 0.017744 | 0.774705 |
| 50 | Nname,cis-9,10-Epoxystearic acid | 297.2405 | 46.197 | 1.258636 | 0.049108 | 0.821076 |
| 51 | O-Phospho-L-homoserine | 198.0156 | 383.1 | 2.037526 | 0.000125 | 0.668402 |
| 52 | O-Succinyl-L-homoserine | 256.0262 | 34.511 | 1.855419 | 0.001187 | 0.566435 |
| 53 | p-Cresol | 107.0527 | 22.808 | 1.688575 | 0.000322 | 0.398724 |
| 54 | Phenylpropionylglycine | 228.0636 | 370.739 | 1.723862 | 0.000663 | 0.490523 |
| 55 | Phosphoenolpyruvate | 188.9609 | 417.7475 | 1.728803 | 0.006099 | 1.729319 |
| 56 | Primidone | 218.1096 | 306.533 | 1.508488 | 0.044201 | 0.481884 |
| 57 | Prostaglandin A2 | 315.1929 | 71.462 | 1.465326 | 0.018086 | 0.45263 |
| 58 | Rutin | 669.1598 | 246.305 | 1.441052 | 0.029822 | 1.527475 |
| 59 | Saccharin | 242.012 | 172.531 | 1.874425 | 0.002938 | 0.563796 |
| 60 | S-Adenosyl-L-homocysteine | 383.1176 | 281.341 | 1.661942 | 0.007921 | 1.932309 |
| 61 | Salicyluric acid | 194.0441 | 217.392 | 2.048617 | 7.57E-05 | 0.452661 |
| 62 | Sebacic acid | 201.1114 | 296.462 | 1.646508 | 0.040487 | 0.472575 |
| 63 | Stearidonic Acid | 275.1989 | 43.646 | 1.823539 | 0.007017 | 0.701233 |
| 64 | Uracil mustard | 251.0205 | 52.363 | 1.685099 | 0.006249 | 0.456472 |
| 65 | Uridine | 303.0778 | 251.193 | 1.717025 | 0.031477 | 1.757374 |
| 66 | Urocanic acid | 174.992 | 22.955 | 1.64029 | 0.001816 | 0.403186 |
| 67 | Xanthurenic acid | 204.0316 | 165.418 | 1.992869 | 0.000134 | 0.658479 |
| 68 | Zearalenone | 317.143 | 436.4355 | 1.224777 | 0.030914 | 1.948395 |

**Supplementary Table 4.** Significantly differentially expressed metabolites in the kidney of AKI+JPYSF mice *versus* AKI mice in positive ion mode

| NO. | Metabolites | m/z | Rt (min) | VIP | P Value | Fold Change |
| --- | --- | --- | --- | --- | --- | --- |
| 1 | (-)-Riboflavin | 341.1206 | 46.772 | 1.770146 | 0.00513 | 1.440785 |
| 2 | .beta.-Cyano-L-alanine | 175.069 | 203.258 | 1.508837 | 0.028199 | 0.533071 |
| 3 | 2(1H)-Pyridinone | 96.04295 | 61.957 | 1.645728 | 0.017581 | 1.209232 |
| 4 | 25-hydroxyvitamin D3 | 383.326 | 32.93 | 1.702151 | 0.016826 | 1.614243 |
| 5 | 2'-O-methylcytidine | 324.0619 | 24.705 | 1.67371 | 0.011077 | 1.272767 |
| 6 | 3-Butynoic acid | 148.0406 | 182.073 | 1.353438 | 0.038398 | 1.600006 |
| 7 | 3-Hydroxyisovaleric acid | 182.0785 | 417.044 | 1.439246 | 0.025314 | 1.596607 |
| 8 | 4-acetamidobutanoate | 206.1009 | 308.0955 | 1.389392 | 0.037292 | 1.905206 |
| 9 | 4-Hydroxybenzaldehyde | 262.1015 | 355.014 | 1.528601 | 0.017262 | 1.39555 |
| 10 | 4-Pyridoxic acid | 184.0582 | 39.2805 | 2.189313 | 2.55E-05 | 0.246875 |
| 11 | 5-Hydroxymethylcytidine | 256.0918 | 395.5475 | 1.797525 | 0.001338 | 0.600499 |
| 12 | 5-Methylcytidine | 258.1054 | 238.209 | 1.393842 | 0.04821 | 0.687564 |
| 13 | 5-Oxo-ETE | 319.2228 | 45.58 | 1.372429 | 0.036326 | 0.75465 |
| 14 | 7-Oxocholesterol | 401.3363 | 33.513 | 1.685515 | 0.042138 | 1.651071 |
| 15 | Acadesine (Drug) | 259.1006 | 186.1735 | 1.567943 | 0.027019 | 0.463129 |
| 16 | Acetylcholine | 146.1154 | 187.627 | 1.94928 | 0.010203 | 1.603717 |
| 17 | Acetyl-DL-Valine | 319.1907 | 347.067 | 1.443437 | 0.044632 | 0.693551 |
| 18 | Agomelatine | 288.0978 | 308.367 | 1.265271 | 0.042565 | 1.406264 |
| 19 | Ala-Gly | 207.0941 | 308.101 | 1.311736 | 0.042152 | 1.839974 |
| 20 | Anthranilic acid (Vitamin L1) | 137.0442 | 323.4485 | 1.69937 | 0.0314 | 1.426548 |
| 21 | Arachidonoyl ethanolamide | 348.2856 | 35.384 | 1.35729 | 0.042758 | 1.534101 |
| 22 | Arg-Cys | 319.1576 | 437.462 | 1.205747 | 0.049556 | 1.782093 |
| 23 | Arg-Ser | 226.1267 | 310.841 | 1.423634 | 0.019806 | 1.351741 |
| 24 | Betaine | 118.0855 | 256.156 | 1.734456 | 0.020372 | 1.435967 |
| 25 | Betaine aldehyde | 102.0902 | 389.79 | 1.435983 | 0.022324 | 1.234785 |
| 26 | Bethanechol cation | 238.0425 | 372.842 | 1.771737 | 0.005414 | 0.728579 |
| 27 | Corticosterone | 347.2177 | 40.983 | 1.708767 | 0.00983 | 0.630962 |
| 28 | Crotonic acid | 190.1046 | 204.709 | 1.840156 | 0.009469 | 0.396238 |
| 29 | Cytidine 5'-monophosphate (CMP) | 346.037 | 419.771 | 1.404814 | 0.039041 | 0.797169 |
| 30 | Daidzein | 255.0617 | 46.3355 | 1.250762 | 0.03936 | 0.663659 |
| 31 | Deoxyinosine | 294.1162 | 83.963 | 1.85659 | 0.012443 | 1.74531 |
| 32 | DL-2-Aminoadipic acid | 206.0374 | 380.4435 | 1.781562 | 0.006914 | 0.519728 |
| 33 | Duloxetine | 262.1076 | 87.7595 | 1.784555 | 0.006229 | 0.496278 |
| 34 | Ethyl glucuronide | 240.1076 | 522.947 | 1.688994 | 0.00279 | 0.591225 |
| 35 | Flavin adenine dinucleotide (FAD) | 786.155 | 367.148 | 1.705705 | 0.013993 | 1.192632 |
| 36 | Flavone | 223.0796 | 61.963 | 1.480539 | 0.02527 | 1.34426 |
| 37 | Gln-Asn | 225.0969 | 370.896 | 1.863831 | 0.000141 | 0.628441 |
| 38 | Glycerophosphocholine | 258.1084 | 367.897 | 1.583809 | 0.007734 | 0.842863 |
| 39 | Glycyl-L-leucine | 227.0846 | 370.283 | 1.830815 | 0.003042 | 0.710836 |
| 40 | Guanosine | 284.0967 | 246.584 | 1.546575 | 0.024055 | 1.356613 |
| 41 | Guanosine 5'-monophosphate (GMP) | 386.0513 | 365.871 | 1.427124 | 0.011734 | 0.635483 |
| 42 | His-Pro | 235.1158 | 134.558 | 1.674242 | 0.004317 | 0.51832 |
| 43 | His-Thr | 257.1211 | 320.324 | 1.608331 | 0.011368 | 1.30016 |
| 44 | Homovanillic acid | 382.1422 | 204.837 | 1.417414 | 0.008063 | 0.800161 |
| 45 | Ile-Ala | 279.0473 | 349.384 | 1.850306 | 0.001927 | 0.548737 |
| 46 | Ile-Arg | 288.1996 | 318.044 | 1.355913 | 0.018792 | 0.868368 |
| 47 | Ile-Val | 231.1674 | 180.805 | 1.583316 | 0.023271 | 0.723739 |
| 48 | Inosine | 269.0858 | 204.556 | 1.469009 | 0.047514 | 1.328754 |
| 49 | Isobutyrylglycine | 146.0788 | 197.5755 | 1.472851 | 0.021347 | 0.846514 |
| 50 | Isovaleric acid | 120.1002 | 328.229 | 1.539613 | 0.019554 | 1.483764 |
| 51 | Larixinic Acid | 144.0634 | 242.939 | 1.548087 | 0.029903 | 1.514988 |
| 52 | L-Carnitine | 162.1109 | 372.1925 | 1.900654 | 0.002057 | 1.328559 |
| 53 | L-Glutamate | 148.0582 | 425.662 | 1.244893 | 0.044398 | 1.30412 |
| 54 | L-Saccharopine | 277.1367 | 420.361 | 1.800512 | 0.003035 | 0.51379 |
| 55 | L-Tyrosine | 182.0789 | 350.178 | 1.829695 | 0.028264 | 2.875503 |
| 56 | L-Valine | 118.0843 | 281.342 | 1.686696 | 0.010109 | 1.120532 |
| 57 | Lys-Cys | 214.1048 | 421.024 | 1.590068 | 0.015274 | 0.524719 |
| 58 | Lys-Gly | 280.0502 | 349.378 | 1.618872 | 0.009535 | 0.636987 |
| 59 | Lys-His | 328.1393 | 205.349 | 1.585483 | 0.030447 | 1.145453 |
| 60 | Lys-Leu | 304.1583 | 372.847 | 1.828718 | 0.003267 | 1.671618 |
| 61 | Lys-Phe | 316.1577 | 470.7 | 1.532668 | 0.018705 | 1.37997 |
| 62 | Metronidazole | 232.0889 | 308.19 | 1.3545 | 0.036411 | 1.421742 |
| 63 | MG(18:2(9Z,12Z)/0:0/0:0)[rac] | 355.2798 | 34.163 | 1.988087 | 0.001351 | 0.777648 |
| 64 | Monoethylglycylxylidide (MEGX) | 245.1052 | 312.711 | 1.407833 | 0.02088 | 1.333304 |
| 65 | N,N-Bis(2-hydroxyethyl)glycine | 224.1103 | 402.722 | 1.549814 | 0.036883 | 1.32259 |
| 66 | N.alpha.-Acetyl-L-lysine | 189.1208 | 370.274 | 1.685064 | 0.020395 | 1.452062 |
| 67 | N-Acetyl-D-galactosamine | 336.0838 | 396.011 | 1.809417 | 0.000907 | 0.619793 |
| 68 | N-Acetyl-L-aspartic acid | 236.0767 | 354.497 | 1.229165 | 0.046411 | 1.319218 |
| 69 | NG,NG-dimethyl-L-arginine(ADMA) | 203.1479 | 487.901 | 1.547637 | 0.025669 | 1.197961 |
| 70 | Nitrobenzene | 264.1008 | 25.13 | 1.699553 | 0.003277 | 0.279311 |
| 71 | Nname,Cinoxacin | 263.0693 | 25.2805 | 1.598964 | 0.010767 | 0.407858 |
| 72 | N-Tigloylglycine | 158.0791 | 182.0855 | 1.94774 | 0.001149 | 0.620743 |
| 73 | p-Acetamidophenol (Acetaminophen, Tylenol) | 320.1605 | 437.629 | 1.287807 | 0.041631 | 1.630442 |
| 74 | PC(16:0/16:0) | 756.5438 | 36.815 | 1.601747 | 0.006388 | 1.665238 |
| 75 | Promethazine | 361.0548 | 369.021 | 1.693813 | 0.005784 | 1.269592 |
| 76 | Propoxur | 227.1442 | 273.749 | 1.315943 | 0.04467 | 0.703949 |
| 77 | Pro-Thr | 258.1472 | 256.104 | 1.705651 | 0.017806 | 1.773772 |
| 78 | Pyridoxal (Vitamin B6) | 168.0631 | 98.265 | 1.759756 | 0.007208 | 1.49343 |
| 79 | Pyridoxamine 5'-phosphate | 248.0584 | 25.7945 | 1.728529 | 0.005156 | 0.351223 |
| 80 | Quinolinate | 231.0325 | 26.125 | 1.960376 | 0.000226 | 0.283767 |
| 81 | Salicylic acid | 121.0264 | 32.709 | 1.39192 | 0.048153 | 0.801492 |
| 82 | sn-Glycerol 3-phosphoethanolamine | 216.0606 | 372.847 | 1.900222 | 0.000982 | 0.672133 |
| 83 | Succinate | 119.0333 | 204.634 | 1.489325 | 0.038039 | 1.295269 |
| 84 | Taurine | 126.0211 | 276.38 | 1.635021 | 0.019053 | 1.172539 |
| 85 | Tyr-Asp | 296.0952 | 107.416 | 1.371608 | 0.040713 | 1.452838 |
| 86 | Verapamil | 429.2581 | 32.907 | 1.676587 | 0.004493 | 1.964257 |
| 87 | Xanthurenic acid | 206.0452 | 165.38 | 2.101722 | 7.59E-05 | 0.633721 |
